# Role of ADAM17 in the non-cell autonomous effects of oncogene-induced senescence

**DOI:** 10.1186/s13058-015-0619-7

**Published:** 2015-08-12

**Authors:** Beatriz Morancho, Águeda Martínez-Barriocanal, Josep Villanueva, Joaquín Arribas

**Affiliations:** Preclinical Research Program, Vall d’Hebron Institute of Oncology (VHIO), Psg. Vall d’Hebron 119-129, Barcelona, 08035 Spain; Department of Biochemistry and Molecular Biology, Building M, Campus UAB, Bellaterra (Cerdanyola del Valles), Barcelona, 08193 Spain; Institució Catalana de Recerca i Estudis Avançats (ICREA), Passeig Lluis Companys 23, Barcelona, 08010 Spain

## Abstract

**Introduction:**

Cellular senescence is a terminal cell proliferation arrest that can be triggered by oncogenes. One of the traits of oncogene-induced senescence (OIS) is the so-called senescence-associated secretory phenotype or senescence secretome. Depending on the context, the non-cell autonomous effects of OIS may vary from tumor suppression to promotion of metastasis. Despite being such a physiological and pathologically relevant effector, the mechanisms of generation of the senescence secretome are largely unknown.

**Methods:**

We analyzed by label-free proteomics the secretome of p95HER2-induced senescent cells and compared the levels of the membrane-anchored proteins with their transcript levels. Then, protein and RNA levels of ADAM17 were evaluated by using Western blot and reverse transcription-polymerase chain reaction, its localization by using biotin labeling and immunofluorescence, and its activity by using alkaline phosphatase-tagged substrates. The p95HER2-expressing cell lines, senescent MCF7 and proliferating MCF10A, were analyzed to study ADAM17 regulation. Finally, we knocked down ADAM17 to determine its contribution to the senescence-associated secretome. The effect of this secretome was evaluated in migration assays in vitro and in nude mice by assessing the metastatic ability of orthotopically co-injected non-senescent cells.

**Results:**

Using breast cancer cells expressing p95HER2, a constitutively active fragment of the proto-oncogene HER2 that induces OIS, we show that the extracellular domains of a variety of membrane-bound proteins form part of the senescence secretome. We determine that these proteins are regulated transcriptionally and, in addition, that their shedding is limited by the protease ADAM17. The activity of the sheddase is constrained, at least in part, by the accumulation of cellular cholesterol. The blockade of ADAM17 abrogates several prometastatic effects of the p95HER2-induced senescence secretome, both in vitro and in vivo.

**Conclusions:**

Considering these findings, we conclude that ectodomain shedding is tightly regulated in oncogene-induced senescent cells by integrating transcription of the shedding substrates with limiting ADAM17 activity. The remaining activity of ADAM17 contributes to the non-cell autonomous protumorigenic effects of p95HER2-induced senescent cells. Because ADAM17 is druggable, these results represent an approximation to the pharmacological regulation of the senescence secretome.

**Electronic supplementary material:**

The online version of this article (doi:10.1186/s13058-015-0619-7) contains supplementary material, which is available to authorized users.

## Introduction

Cellular senescence is a terminal cell proliferation arrest characterized by a distinct phenotype. Compared with their proliferating counterparts, senescent cells have enlarged volumes, display a flattened and vacuolated morphology, and express a variety of markers. The most widely used to identify senescent cells is senescence-associated β-galactosidase.

Cellular senescence can be triggered by a variety of stressors, including oncogenes, resulting in what is known as oncogene-induced senescence (OIS) [1]. For example, expression of p95HER2, an oncogenic fragment of the tyrosine kinase receptor HER2, induces OIS in a variety of cell lines [2].

The onset of senescence is characterized by a profound change in the secretome (i.e., all factors secreted by a given cell) that results in the so-called senescence-associated secretory phenotype or senescence secretome [1]. Depending on the context, the senescence secretome has disparate effects. It may promote [3] or impair [4] immune surveillance against tumor cells in the liver and in the prostate, respectively. In fact, senescent cells may be short-lived or long-lived in vivo, in both immunocompetent [3–5] and immunosuppressed [2, 6] mice. Furthermore, the senescence secretome can suppress [7] or promote [8] tumor growth. These results can be rationalized assuming that the potent tumor suppressive effects of senescence can be reversed, particularly in advanced tumors, by modifying the composition of the senescence secretome and, thus, its effects on target cells.

Because the non-cell autonomous effects of senescent cells can suppress or contribute to tumor progression, the up- or downregulation of the senescence secretome could be a therapeutic strategy to treat cancer and perhaps many other diseases related to cellular senescence [1]. Unfortunately, to date, there are no known strategies to regulate the production of the senescence secretome.

The proteolytic release of the extracellular domain of transmembrane proteins is known as ectodomain shedding. This type of limited proteolysis affects a diverse group of functionally unrelated transmembrane proteins, including membrane-anchored growth factors, cytokines, cell adhesion molecules, or transmembrane proteases [9–12]. The proteases that cleave the vast majority of these transmembrane proteins are the metalloprotease disintegrins ADAM17 (also known as tumor necrosis factor-alpha-converting enzyme) or ADAM10 or both (reviewed in [13]).

Some components frequently secreted by senescent cells, such as transmembrane epidermal growth factor (EGF)-like growth factors, are generated through ectodomain shedding. However, the contribution of ectodomain shedding to the senescence secretome remains largely unexplored. Although ADAM17 has been recently shown to be active in senescent cells [14], its regulation or functional importance during senescence is unknown.

Here, we show that approximately 10 % of the components of the secretome of p95HER2-induced senescent cells are generated through the shedding of the ectodomains of membrane-anchored proteins. The main mechanism regulating the release of these ectodomains is the transcriptional regulation of the membrane-anchored precursors. Functional analysis shows that ADAM17 plays a major role in these cleavages. However, although ADAM17 protein levels increase during p95HER2-induced OIS, the activity of the metalloprotease does not increase, and this is likely because of the accumulation of cholesterol, a negative regulator of ADAM17, in senescent cells. Finally, we show that ADAM17 activity is required for several non-cell autonomous protumorigenic and prometastatic effects of p95HER2-induced senescent cells. Because the activity of ADAM17 can be pharmacologically downregulated, these results indicate that inhibition of this metalloprotease could be a means to target the undesired non-cell autonomous effects of cellular senescence.

## Methods

### Reagents

Doxycycline (Doxy.), phorbol myristate acetate, biotin, 1-10-phenanthroline, methyl-beta-cyclodextrin (MβCD), insulin, EGF, and hydrocortisone were from Sigma-Aldrich (St. Louis, MO, USA). Batimastat (BB94) was from Merck (Schwalbach, Germany).

### Antibodies

Rabbit anti-phospho ERK (T202/Y204; #4370), anti-ERK (#9102), anti-phospho Akt (S473; #9271), anti-Akt (#9272), anti-phospho EGF receptor (EGFR) (Y1068; #3777), and anti-EGFR (#4267) were from Cell Signaling Technology (Danvers, MA, USA). Mouse anti-EpCAM (#sc-25308) was from Santa Cruz Biotechnologies (Santa Cruz, CA, USA), and mouse anti-pan actin (#MA5-11869) was from Thermo Scientific (Lafayette, CO, USA). Mouse anti-APP (#MAB348) and rabbit anti-ADAM17 (#AB19027) were from Millipore (Billerica, MA, USA). Rabbit anti-ADAM17 (#ab39162), rabbit anti-ADAM10 (#ab1997), goat anti-Met receptor (#ab10728), and mouse anti-glucose-6-phosphate (GPI) (#ab66340) antibodies were from Abcam (Cambridge, MA, USA). Rabbit anti-DDR1 (#10730) was from Sino Biological Inc. (Beijing, PR China), rabbit anti-GAPDH (#2275-PC-1) was from Trevigen (Gaithersburg, MD, USA), and mouse anti-HER2 (#MU-134-UC) was from BioGenex (San Ramon, CA, USA). Mouse anti-EphB4 (#AF446) was from R&D Systems (Minneapolis, MN, USA). Fluorochrome-conjugated antibodies were from Molecular Probes (Life Technologies, Grand Island, NY, USA).

### Transcriptomic and proteomic analyses

Transcripts and secretome analysis were performed as described in [15] and [2], respectively. GeneChip expression probe array Gene Expression Omnibus reference number is GSE68256. Heatmap hierarchical clustering was performed as in [16].

### Plasmids

The expression vectors encoding alkaline phosphatase (AP)-tagged amphiregulin (AP-Areg), transforming growth factor-alpha (AP-TGF-α), and betacellulin (AP-BTC) were a kind gift from Shigeki Higashiyama (Department of Biochemistry and Molecular Genetics, Ehime University Graduate School of Medicine, Japan). Subcloning in the lentiviral vector pLex (Thermo Scientific) was performed by conventional molecular biology techniques. pGIPZ-based shRNA vectors targeting ADAM17 or non-silencing control were from Thermo Scientific. p95HER2 in pENTR1Dual (Invitrogen, Paisley, UK) was transferred into pINDUCER20 using LR Clonase II (Invitrogen). For control pINDUCER20 (pINDUCER20-Empty), cloramphenicol and ccdB genes were removed from pENTR1A Dual (5-SalI and 3’-XhoI) and the minimal MCS was transferred to pINDUCER20. pINDUCER20 was kindly provided by Stephen J Elledge (Howard Hughes Medical Institute, Chevy Chase, MD, USA) [17].

### Cell culture and infections

MCF7, MCF7 Tet-Off p95HER2, and MDA-MB-231-Luc were maintained as previously described [2]. MCF10A was maintained in Dulbecco’s modified Eagle’s medium,/F-12, 10 % fetal bovine serum (FBS), 4 mmol/l L-glutamine (all from Gibco, Carlsbad, CA, USA), 9 μg/ml insulin, 0.5 μg/ml hydrocortisone, and 18 ng/ml EGF. MCF7 Tet-Off p95HER2 AP-Areg, AP-TGF-α, AP-BTC, shControl, and shADAM17 were generated by infecting MCF7 Tet-Off p95HER2 with the lentiviral particles obtained transfecting HEK293T cells with the plasmids described above (calcium phosphate method). Viral supernatant was applied to the cells in the presence of 8 μg/ml polybrene, and stable clones were selected with 1 μg/ml puromycin. MCF10A was infected with pINDUCER20-p95HER2 by using the same protocol, and selection was performed with 200 μg/mL G418. MCF10A infection with lentiviral vectors expressing AP-Areg and AP-TGF-α was performed with the plasmids and method indicated above.

### Protein extraction and immunoblotting

Cells were lysed in 20 mM Tris-HCl pH 7.4, 137 mM NaCl, 2 mM EDTA pH 8.0, 10 % glycerol, 1 % NP-40, protease inhibitor cocktail (Roche, Penzberg, Germany), 1.3 mM sodium orthovanadate, 10 mM 1-10-phenanthroline, and 1 μM BB94. Cell lysates were quantified by using the Pierce BCA Protein Assay kit (Pierce, Rockford, IL, USA), and equal amounts of protein were separated on SDS-polyacrylamide gels and transferred onto nitrocellulose membranes. These were blocked with 5 % bovine serum albumin or skim milk in TBS-Tween 0.1 % and then incubated with primary antibodies, and bound antibodies were detected with the appropriate peroxidase-conjugated secondary antibodies (Amersham GE Healthcare, Piscataway, NJ, USA) by using the ECL detection system (Millipore). To detect ADAM17, the same amount of protein from cell lysates was concentrated with wheat germ agglutinin (WGA)-agarose beads (Vector Laboratories, Peterborough, UK) for 2 h at 4 °C. Proteins were eluted directly in SDS-polyacrylamide gel electrophoresis loading buffer.

Determination of proteins in the collected conditioned media was performed following the same protocol after concentrating 100× the samples by using centricons (Amicon Ultracel 3K; Millipore). Densitometric quantification was performed by using ImageJ software.

### Enzyme-linked immunosorbent assay

Cells were plated and the next day were washed twice with 1X phosphate-buffered saline (PBS), and medium was changed to serum-free medium with L-glutamine. Conditioned media were collected 48 h later, spun down at 200×*g* for 5 min, and transferred to clean tubes. Amphiregulin was determined in accordance with the instructions of the manufacturer (RayBiotech, Norcross, GA, USA).

### mRNA expression

RNA was isolated by using an RNeasy Mini kit (Qiagen, Hilden, Germany) and reverse-transcribed by using a High Capacity cDNA Reverse Transcription Kit (Applied Biosystems, Weiterstadt, Germany) in accordance with the instructions of the manufacturers. Quantitative reverse transcription-polymerase chain reaction was performed by using Taqman primers (Applied Biosystems) for ADAM17 (Hs01041915_m1), ADAM10 (Hs00153853_m1), and GAPDH (Hs03929097_g1).

### Biotin labeling

Cells were washed three times with ice-cold 1X PBS pH 8.0 and labeled with 1 mg/ml sulfo-NHS-LC-biotin (Pierce) in 1X PBS pH 8.0 gently shaking for 1 h at 4 °C. Biotin was inactivated by washing with 50 mM Tris pH 7.4, and cells were extensively washed with 1X PBS. Then cells were harvested and lysed, and immunoprecipitation was performed by using an anti-ADAM17 antibody or a control IgG. Immunoprecipitates were analyzed by Western blotting by using streptavidin-POD conjugate (Roche) or anti-ADAM17 antibody (Abcam).

### Confocal microscopy

Cells were seeded on glass coverslips, washed with 1X PBS, fixed with 4 % formaldehyde for 20 min, and permeabilized with 0.2 % Triton X-100 for 10 min. 1X PBS containing 1 % bovine serum albumin, 0.1 % saponin, and 0.02 % NaN_3_ was used for blocking (1 h), primary antibody binding (2 h), and secondary fluorochrome-conjugated antibody binding (40 min in dark). Preparations were mounted by using Vectashield with DAPI (4’,6’-diamidino-2-phenylindole) (Vector Laboratories). All procedures were performed at room temperature. Images were captured by using an Olympus FV1000 confocal microscope (Olympus Corporation, Tokyo, Japan).

### Ectodomain shedding assay

MCF7 Tet-Off p95HER2 cells were induced for 5 days and replated at the same cell concentration. Next day, cells were washed with Opti-MEM (Invitrogen) without serum or growth factors, and the medium was replaced 1 h later with fresh Opti-MEM with or without the indicated stimuli. Cells and supernatants were harvested at the designated time points, and AP activity was measured at an absorbance of 405 nm after incubation with the AP substrate 4-nitrophenyl phosphate (Sigma-Aldrich). At least three wells per condition were performed, and the ratio between the AP activity in the supernatant and the cell lysate plus supernatant was calculated. The fold increase in the ratio of AP activity relative to the control is shown.

### Proliferation assays

Proliferation was analyzed by cell counting. After trypsinization, viable cells determined by trypan blue dye exclusion were counted on a Neubauer chamber.

### Senescence-associated β-galactosidase activity

Cells were plated on coverslips and analyzed by using a senescence β-galactosidase staining kit (Cell Signaling Technology) in accordance with the indications of the manufacturer.

### Filipin staining

Cells were washed three times with 1X PBS and fixed with 3 % paraformaldehyde for 1 h at room temperature. Washes were repeated, and cells were incubated with 1.5 mg/ml glycine in 1X PBS for 10 minutes. Staining was performed for 2 h with 0.05 mg/ml filipin complex (Sigma-Aldrich) in 1X PBS/10 % FBS. After the cells were rinsed three times with 1X PBS, they were analyzed by fluorescence microscopy (BX61 Olympus).

### Migration assays

Cell migration was determined with 24-well format Boyden chambers containing FluoroBlok PET membranes with 8-μm pores (BD Biosciences, Heidelberg, Germany). Conditioned media were obtained after incubating cells with serum-free medium with glutamine for 48 h, spun down, and concentrated 100 times by using centricons (Amicon Ultracel 3K). Concentrated conditioned media were diluted 10 times in serum-free medium and added to the bottom part of the Boyden chambers. MDA-MB-231 Luc or MCF7 cells were plated in the upper chambers and migration ability was evaluated 24 or 48 h later, respectively, by staining the cells with 1 μM SYTO-9 (Life Technologies) and counting them on the underside of the filters by using fluorescence microscopy.

### Mouse model of breast cancer metastasis

MCF7 Tet-Off p95HER2 cells expressing a short hairpin control or targeting ADAM17 were co-injected with MDA-MB-231 Luc cells into the right flanks of 6- to 8-week-old female BALB/c athymic mice (Charles River Laboratories, Paris, France) in addition to a 17β-estradiol pellet (Innovative Research of America, Sarasota, FL, USA). The expression of p95HER2 was repressed by adding doxycycline (50 mg/kg per day) to the drinking water until tumors were about 100 mm^3^. Tumor xenografts were measured with calipers every 3 days, and tumor volume was determined by using the formula: (length × width^2^) × (pi/6). Tumors were resected when they reached 300 mm^3^, and metastatic colonization was monitored by in vivo bioluminescence imaging with the IVIS-200 imaging system (PerkinElmer, Waltham, MA, USA). At the end of the experiment, animals were anesthetized with a 1.5 % isofluorane-air mixture and were sacrificed by cervical dislocation. Mice were maintained and treated in accordance with institutional guidelines of Vall d’Hebron University Hospital Care and Use Committee.

### Statistics

Data are presented as average ± standard deviation and were analyzed by the two-sided Student *t* test. Results were considered to be statistically significant at *P* value of less than 0.05. All statistical analyses were conducted by using the GraphPad Prism 5 Statistical Software (GraphPad Software, Inc., La Jolla, CA, USA).

## Results

### Shedding of transmembrane molecules during p95HER2-induced senescence

Expression of p95HER2 in different breast cancer cell lines leads to OIS ([2] and Additional file 1: Figure. S1a). Analysis of the media conditioned by control cells or p95HER2-induced senescent cells by quantitative label-free proteomics confirmed the profound change in the secretome composition characteristic of OIS (Additional file 1: Figure. S1b).

The analysis of the extracellular proteins identified showed that the majority (79 %) contained neither signal peptide nor transmembrane domain (Additional file 1: Fig. S1c and Additional file 2: Table S1, Additional file 3: Table S2 and Additional file 4: Table S3), indicating that they are secreted through exosomes or unknown secretory mechanisms; 10 % corresponded to proteins that contained a signal peptide but not a transmembrane or GPI domains (Additional file 1: Figure. S1c and Additional file 4: Table S3), indicating that they are released to the extracellular media through the canonical secretory pathway. Finally, 11 % of the proteins were predicted to be membrane-anchored (Additional file 1: Figure. S1c and Additional file 4: Table S3), opening the possibility that they are secreted through ectodomain shedding. Confirming this possibility, sequence alignment showed that virtually all the peptides identified by mass spectrometry from selected transmembrane proteins corresponded to extracellular domains (Additional file 5: Figure. S2). We chose these proteins because, compared with other transmembrane proteins, they have long intracellular domains; identification of peptides corresponding to the intracellular domains would indicate that they are released through exosomes or other mechanisms. We concluded that approximately 10 % of the components of the p95HER2-induced senescence secretome are generated through ectodomain shedding.

### Regulation of the sheddome of p95HER2-induced senescent cells

**Fig. 1:**
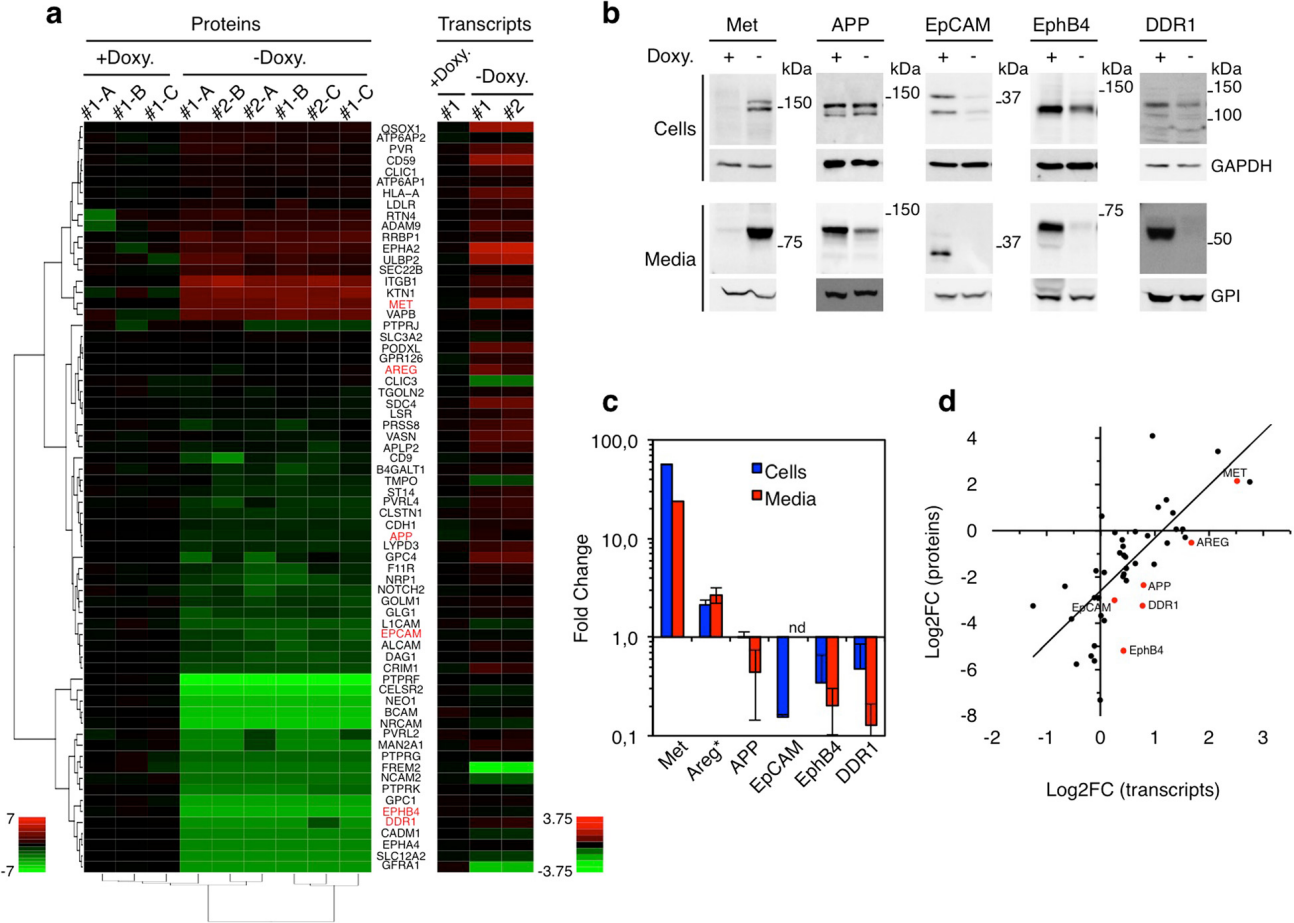
Protein ectodomain shedding during p95HER2-induced senescence. **a** *Left*, MCF7 Tet-Off p95HER2 cells were treated with or without doxycycline as indicated in two independent experiments (#1 and #2). The extracellular media were analyzed by label-free quantitative proteomics in triplicate (**A**-**C**) (Additional file 1: Figure. S1). The heatmap shows log_2_FC of the normalized levels of soluble ectodomains. *Right*, transcriptomic analysis of the same cells treated as in left panel (**a**). **b** MCF7 Tet-Off p95HER2 cells were treated with or without doxycycline for 7 days. Conditioned media from the last two days and cell lysates were analyzed by Western blotting as indicated. **c** Quantitative results from at least three independent experiments performed as in (**b**) were expressed as average ± standard deviation. Note that the levels of Areg were determined by enzyme-linked immunosorbent assay. **d** log_2_FC of the ectodomain levels and the log_2_FC of their corresponding transcripts levels, determined as described in (**a**), are represented. Linear regression line is shown (R^2^ = 0.528); *P* <0.001 using the Spearman correlation coefficient. *APP* amyloid precursor protein, *Areg* amphiregulin, *EpCAM* epithelial cell adhesion molecule, Ephrin type-B receptor 4, *DDR1* discoidin domain receptor family, member 1, *GAPDH* glyceraldehyde 3-phosphate dehydrogenase

Proteomic analysis of membrane-anchored proteins showed the profound change in the sheddome (i.e., the part of the secretome generated by ectodomain shedding) produced by p95HER2-induced senescent cells (Fig. 1a, Proteins).

Analysis by Western blot of several randomly chosen proteins confirmed the expected full-length species and shed ectodomains in cell lysates and conditioned media, respectively (Fig. 1b and Additional file 6: Table S4). Confirming the proteomic analysis, compared with control proliferating cells, the levels of the soluble extracellular domains of the tyrosine kinase receptor Met or the EGF-like growth factor Areg increased during p95HER2-induced senescence. In contrast, the extracellular domains of the amyloid precursor protein (APP), the cell adhesion molecule EpCAM, or the tyrosine kinase receptors DDR1 and EphB4 decreased (Fig. 1b, c).

The increased levels of the soluble ectodomains of Met and Areg were concomitant with increased levels of the cell-associated full-length proteins (Fig. 1b, c). Conversely, the decrease in the levels of the soluble ectodomains of DDR1, EphB4, and EpCAM paralleled those of their full-length cell-associated counterparts. In the case of APP, the levels of the transmembrane protein did not change; however, the levels of shedding decreased (Fig. 1b, c), indicating that, in senescent cells, this substrate is less accessible to the metalloproteases that cleave its ectodomain or, alternatively, that these metalloproteases are inhibited.

The transcriptomic analysis of the factors detected though proteomics showed a direct correlation between the levels of shed ectodomains and those of their corresponding mRNAs (Fig. 1a, Transcripts and Fig. 1d). We concluded that, during OIS, ectodomain shedding is regulated largely through the transcriptional control of shedding substrates.

### ADAM17 expression in p95HER2-induced senescent cells

ADAM17 cleaves Areg [18], APP [19], DDR1 [20], EphB4 [21], Met [22], and EpCAM [23]. Therefore, we analyzed the levels of ADAM17 during the onset of p95HER2-induced senescence.

**Fig. 2:**
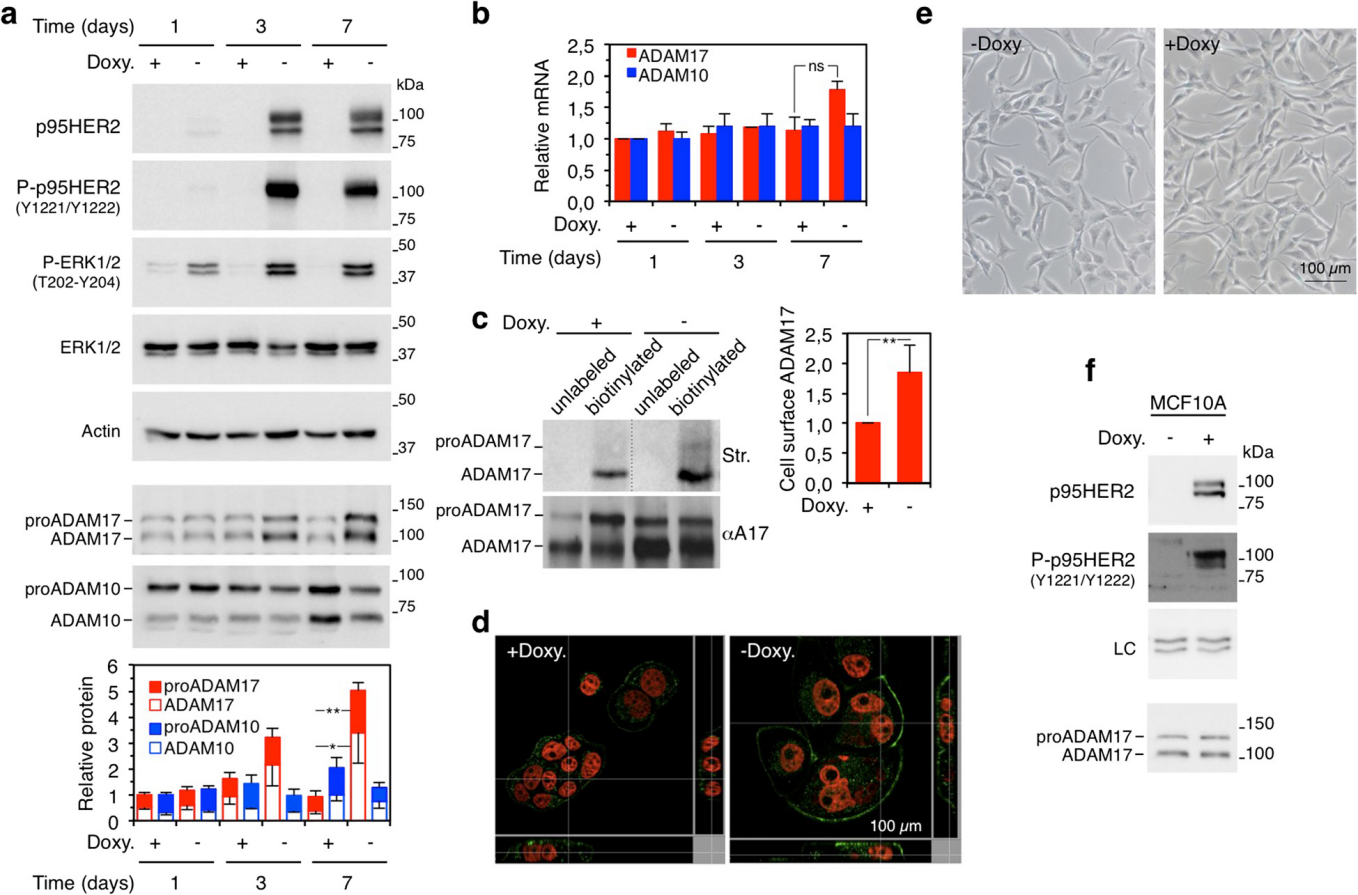
ADAM17 levels during oncogene-induced senescence. **a** MCF7 Tet-Off p95HER2 cells were cultured in the presence or absence of doxycycline and harvested at the specified time points. Then cells were lysed and cell lysates were analyzed by Western blotting with the indicated antibodies. The densitometric quantification of the pro- and mature forms of ADAM17 and ADAM10 was performed in four independent experiments and is expressed as average ± standard deviation. *P <0.05, **P <0.01 using the Student’s *t* test. **b** Relative ADAM17 and ADAM10 mRNA expression was determined by reverse transcription-polymerase chain reaction in the same cells as in (**a**). Data were normalized to 18S mRNA. Averages and standard deviations of three independent experiments are represented. Differences are not significant statistically (ns). **c** MCF7 Tet-Off p95HER2 cells were cultured in the presence or absence of doxycycline for 7 days and incubated with phosphate-buffered saline (unlabeled) or sulfo-NHS-LC-biotin (biotinylated) as described in the Methods section. Then cells were lysed and cell lysates were incubated with anti-ADAM17 antibody and immunoprecipitates were analyzed by Western blotting by using streptavidin-peroxidase (Str.) or anti-ADAM17 (αA17). The densitometric quantification of six independent experiments is expressed as average ± standard deviation. **P <0.01 using the Student’s *t* test. **d** Representative immunofluorescence of ADAM17 (*green*) and DAPI (4’,6’-diamidino-2-phenylindole) counterstaining (nuclei, *red*) in MCF7 Tet-Off p95HER2 cells cultured with or without doxycycline. *Bottom* and *left* panels in each image represent Z-stack integration in the X and Y dimensions, respectively. **e** MCF10A Tet-On p95HER2 cells were cultured for 7 days without or with doxycycline. Then senescence-associated β-galactosidase was performed. Representative bright-field images are shown. **f** The same cells as in (**e**) were lysed and cell lysates were analyzed by Western blot with the indicated antibodies. *Doxy* doxycycline

Expression of p95HER2 during 2 days results in the irreversible onset of senescence, and after 5–7 days the cells display the full senescence phenotype [2]. Concomitantly, the levels of total ADAM17 increased (Fig. 2a, bottom panels). ADAM17 is synthesized as a zymogen (proADAM17), which contains a pro-domain that inhibits the metalloprotease active site. During transport through the trans-Golgi network, furin-like pro-protein convertases remove the pro-domain generating mature ADAM17 [24]. The increase in the levels of the metalloprotease (Fig. 2a, bottom panels) is largely post-transcriptional (Fig. 2b). Analysis of its subcellular distribution by means of biotinylation of intact cells and immunofluorescence showed that ADAM17 is predominantly intracellular in proliferating cells and that it accumulates at the cell surface in p95HER2-induced senescent cells (Fig. 2c, d). ADAM10 also participates in ectodomain shedding, and some of the substrates of ADAM17, such as APP, are also cleaved by ADAM10 [13]. However, ADAM10 protein was not upregulated during p95HER2-induced senescent cells; in fact, relative to control cells, p95HER2-induced senescent cells showed a slight but reproducible downmodulation of ADAM10 which could not be explained by a reduction in its cognate transcript (Fig. 2a, b).

To determine whether the upregulation of ADAM17 is the result of the activity of p95HER2 independently of the senescence status, we analyzed MCF10A cells. Rather than inducing OIS (Fig. 2e), expression of p95HER2 in this immortalized non-transformed mammary epithelium cell line, accelerates proliferation [2]. The levels of ADAM17 remained unchanged in MCF10A expressing p95HER2 (Fig. 2f), indicating that overexpression of the metalloprotease is likely linked to OIS rather than to the mere expression of the constitutively active fragment of HER2. We concluded that the levels of ADAM17 are upregulated during p95HER2-induced cellular senescence.

### Role of ADAM17 in the shedding of different components of the senescence secretome

**Fig. 3:**
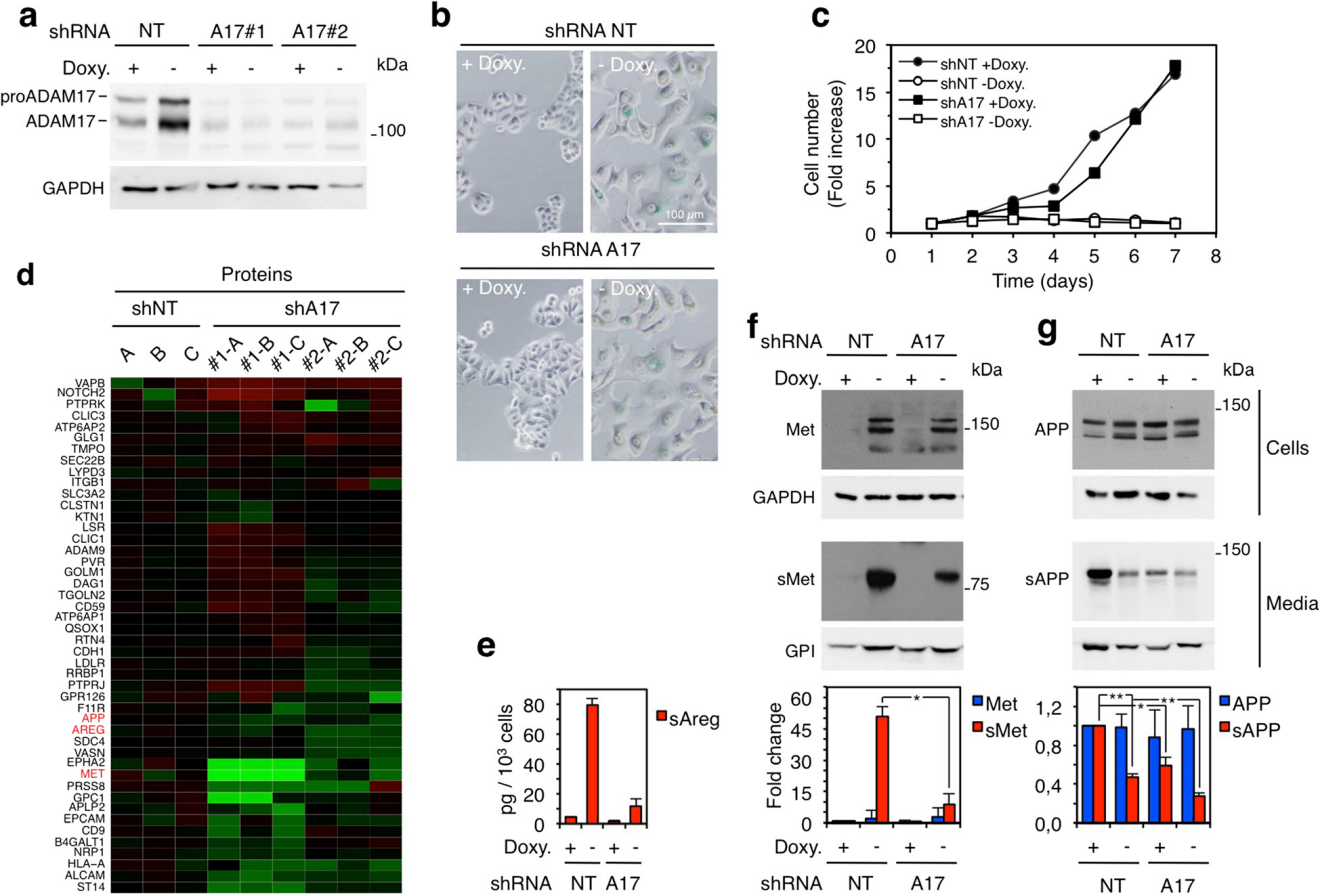
Contribution of ADAM17 to the p95HER2-induced senescence secretome. **a** MCF7 Tet-Off p95HER2 cells constitutively expressing a control shRNA (NT, non-targeting) or two independent shRNAs targeting ADAM17 (A17#1 and A17#2) were cultured with or without doxycycline for 7 days. Cells were then harvested and lysed, and cell lysates were analyzed by Western blotting with the indicated antibodies. **b** Senescence-associated β-galactosidase was analyzed in the same cells as in (**a**). Representative bright-field images are shown. **c** The same cells as in (**a**) were plated with or without doxycycline and counted at the indicated time points. The results represent averages of two independent determinations. **d** The conditioned media of the same cells as in (**a**), treated without doxycycline, were analyzed by label-free quantitative proteomics in triplicate (**a**-**c**). The heatmap shows log_2_FC of the normalized levels of soluble ectodomains. **e**-**g** The same cells as in (**a**) were treated with or without doxycycline for 7 days. Conditioned media from the last two days and cell lysates were analyzed by enzyme-linked immunosorbent assay (**e**) or Western blotting (**f**, **g**) as indicated. Quantification of at least three independent experiments is represented as average and standard deviation. These results were reproduced by using the second stable cell line expressing an independent shRNA targeting ADAM17 (data not shown). *Doxy* doxycycline

To determine whether ADAM17 plays a role in the shedding of transmembrane proteins during OIS, we knocked it down from the MCF7 Tet-Off p95HER2 cells. We used two independent shRNAs targeting ADAM17 to generate two cell lines (Fig. 3a). Although we observed similar results with both cell lines, for simplicity, except in Fig. 3d, we show the results obtained with only one of them. The downmodulation of the protease did not affect the morphology or the levels of SAβG in p95HER2-induced senescent cells (Fig. 3b). The proliferation of control cells or the inhibition of proliferation induced by p95HER2 expression was also unaffected (Fig. 3c).

In a proteomic analysis of the media conditioned by p95HER2-induced senescent cells expressing control shRNAs or the shRNAs targeting ADAM17, we quantified the soluble ectodomains of 68 % (46 out of 68) of the membrane-anchored proteins shown in Fig. 1b. The levels of 37 % of these ectodomains (17 out of 46) decreased in the media conditioned by the ADAM17 knockdown cell lines (Fig. 3d and Additional file 7: Table S5), showing that the metalloprotease participates in the cleavage of the corresponding membrane-anchored factors.

To validate these results, we showed that, in p95HER2-induced senescent cells, the knockdown of ADAM17 resulted in an approximately 80 % reduction of the shedding of Areg and Met (Fig. 3d, e and f).

In agreement with the results shown in Fig. 1c, compared with that in control proliferating cells, the shedding of APP was reduced in p95HER2-induced senescent cells (Fig. 3g). Confirming the result of the proteomic analysis (Fig. 3d), in ADAM17 knockdown senescent cells, the shedding of APP was further reduced (Fig. 3g).

Collectively, these results show that ADAM17 contributes to protein ectodomain shedding during OIS. We estimate that this metalloprotease contributes to the cleavage of approximately one third of the membrane-anchored proteins that undergo ectodomain shedding during OIS.

### ADAM17 activity in p95HER2-induced senescent cells

Although the results in Fig. 3 clearly show a role of ADAM17 in the generation of the senescence secretome, the inhibition of the shedding of APP during p95HER2-induced senescence (Figs. 1b, c and 3g), despite the increase of ADAM17 levels (Fig. 2a), indicates that the activity of the metalloprotease may be partially inhibited in p95HER2-induced senescent cells.

**Fig. 4:**
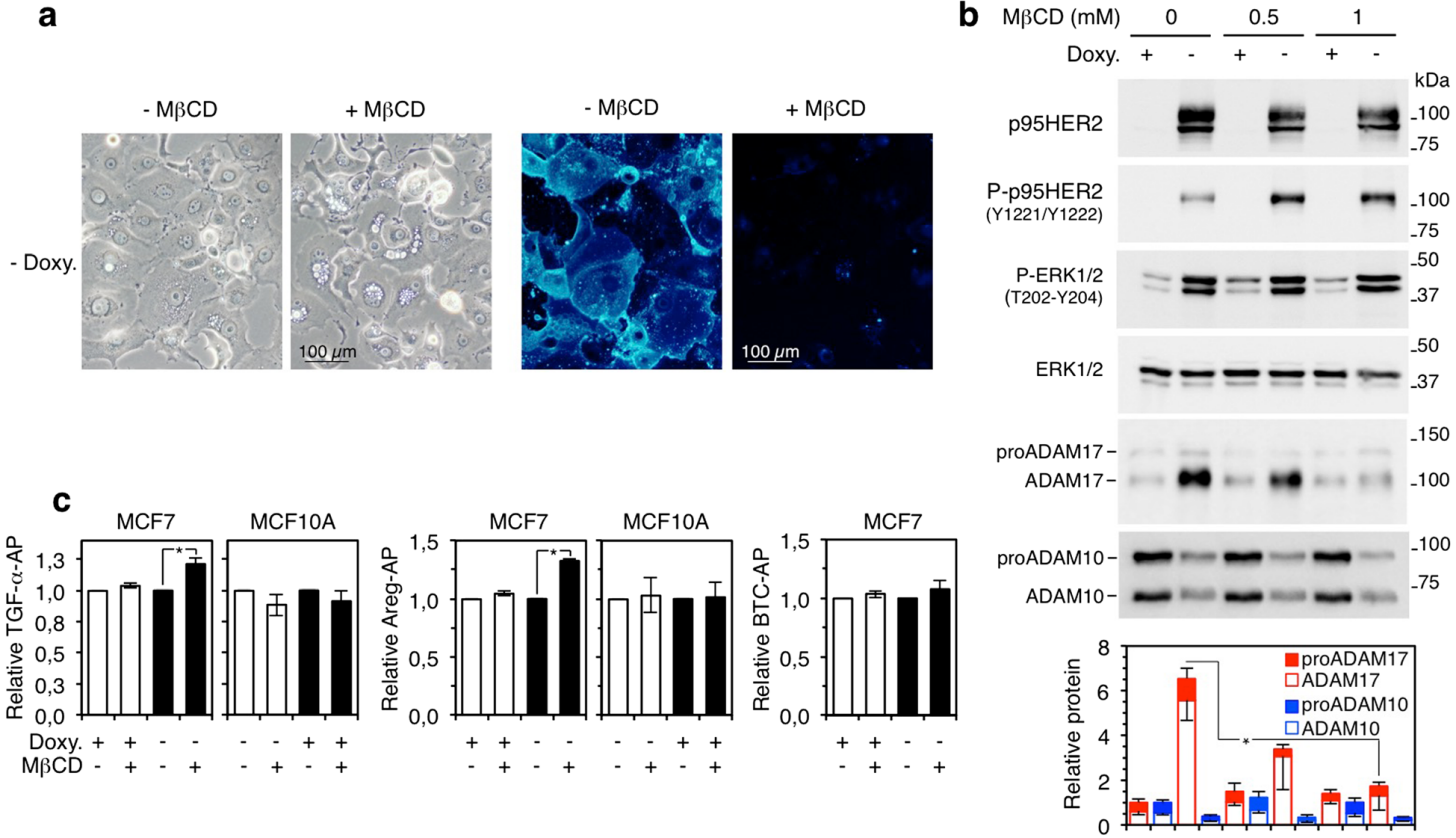
Effect of cholesterol depletion on the protein levels and activity of ADAM17 in p95HER2-induced senescent cells. **a** MCF7 Tet-Off p95HER2 cells were cultured with or without doxycycline for 5 days and further incubated in serum-free media with or without 1 mM MβCD for 2 additional days. Then cells were stained with filipin. Representative bright-field and filipin staining images are shown. **b** The same cells as in (**a**) were treated with different concentrations of MβCD. Control and treated cells were harvested and lysed, and cell lysates were analyzed by Western blot with the indicated antibodies. The densitometric quantification of three independent experiments is expressed as average ± standard deviation. *P <0.05 using the two-sided Student’s *t* test. **c** MCF7 Tet-Off p95HER2 or MCF10A Tet-On p95HER2 cells expressing AP-tagged TGF-α (*left panels*), Areg (*middle panels*), or BTC (*right panel*) were treated with or without doxycycline and MβCD or vehicle for 48 h as indicated. AP was quantified in serum-free conditioned media and cell lysates. Data shown represent the averages and standard deviations of three independent experiments. *P < 0.05 using the two-sided Student’s *t* test. *AP* alkaline phosphatase, *BTC* betacellulin, *Doxy* doxycycline, *MβCD* methyl-beta-cyclodextrin, *TGF* transforming growth factor

Previous reports have shown that ADAM17 is inhibited by high cholesterol levels [25–30]. Senescent cells tend to accumulate cholesterol [31]; in fact, it has been previously shown that HER2 (NeuT)-induced senescence results in a marked accumulation of cellular cholesterol [32]. Thus, we reasoned that the high levels of cholesterol in p95HER2-induced senescent cells may lead to the accumulation of partially inactive ADAM17. As expected, treatment with MβCD, a compound that selectively extracts membrane cholesterol, reduced the levels of cholesterol in p95HER2-senescent cells (Fig. 4a).

MβCD did not affect the expression of p95HER2 or its signaling ability (Fig. 4b); however, it completely prevented the increase in ADAM17 protein levels that occurs during OIS. In contrast, the levels of ADAM10 were largely unaffected by the same treatment (Fig. 4b, bottom panels).

We directly monitored the effect of cholesterol depletion on the activity of ADAM17 using vectors encoding the transmembrane growth factors proTGF-α and proAreg fused to AP. Both EGF-like transmembrane growth factors are cleaved almost exclusively by ADAM17 in different cell types [18, 33], and the AP moiety allows accurate quantitation of shedding [18]. As previously shown [25–30], short-term treatment with MβCD activates ectodomain shedding (Additional file 8: Figure. S3a); however, at longer time-points (48 h), MβCD had little or no effect on the shedding of these factors in control proliferating cells, whether they express p95HER2 or not (Fig. 4c). But consistently with an inhibitory effect of cholesterol, MβCD induced a significant increase in the shedding of both AP-tagged growth factors only in p95HER2-induced senescent cells (Fig. 4c). The shedding of betacellulin (BTC), a substrate of ADAM10 [18], was also unaffected by the treatment with MβCD, ruling out cholesterol as modulator of the activity of ADAM10 in MCF7 cells (Fig. 4c).

We concluded that the increase in cholesterol content in senescent cells downregulates the activity of ADAM17, but not that of ADAM10, and results in accumulation of partially inactive ADAM17. However, it should be underscored that the remaining ADAM17 activity is responsible for the shedding of a variety of transmembrane molecules during OIS (Fig. 3).

### Role of ADAM17 in non-cell autonomous effects of p95HER2-induced senescence

To analyze the functional importance of ADAM17 activity during OIS, we compared the non-cell autonomous effects of p95HER2-induced senescent cells with those of the same cells knocked down for the metalloprotease on different in vitro and in vivo assays.

Consistently with the production of Areg by senescent cells, incubation of A431 cells, which overexpress the EGFR, with the media conditioned by p95HER2-induced senescent cells increased the levels of phospho-EFGR (Additional file 8: Figure. S3b). Also, as expected, knockdown of the metalloprotease completely inhibited the effect of the senescence secretome on phospho-EGFR levels (Additional file 8: Figure. S3b).

**Fig. 5:**
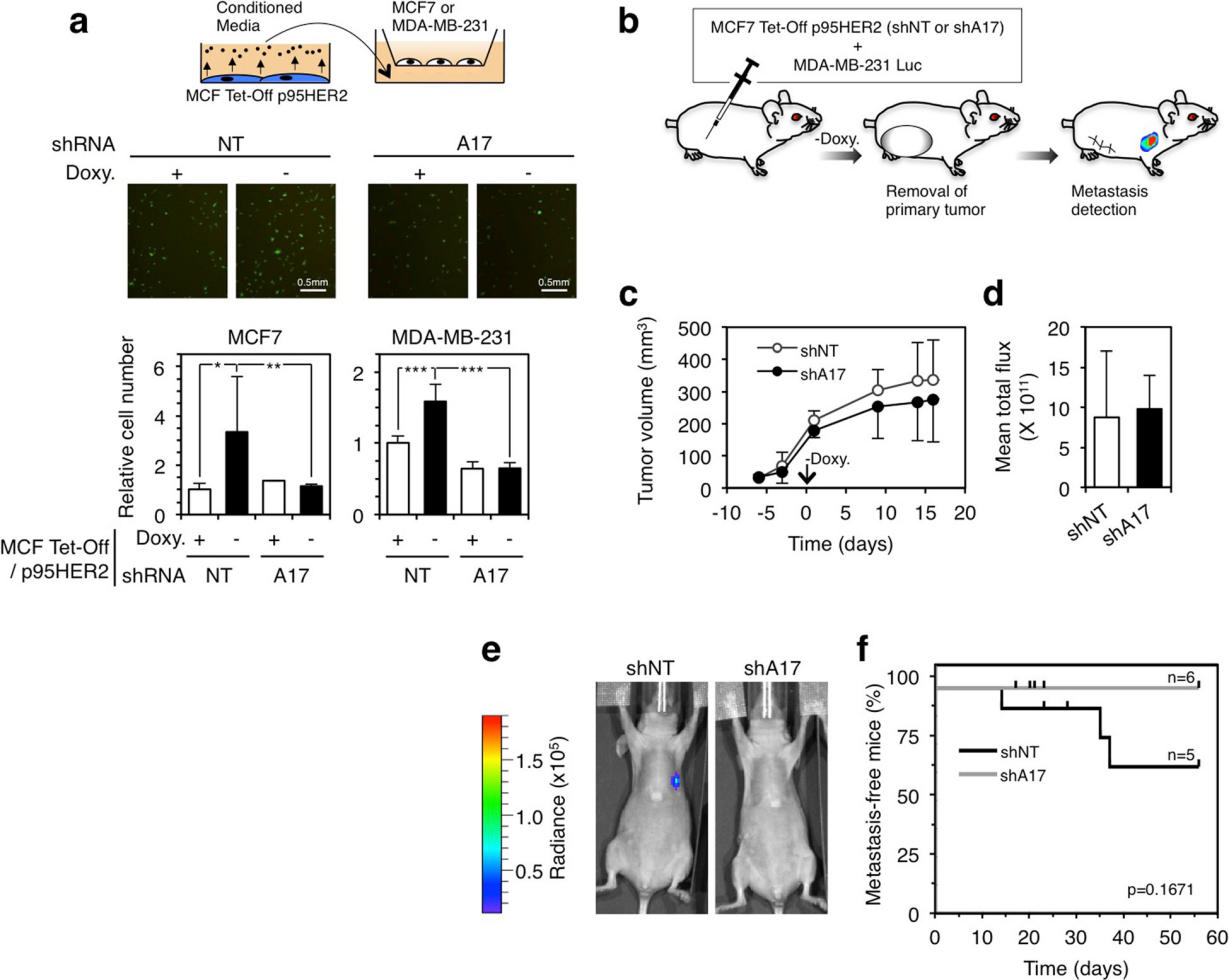
Effect of ADAM17 knockdown on different non-cell autonomous effects of p95HER2-induced senescent cells. **a** Conditioned media from MCF7 Tet-Off p95HER2 shNT or shADAM17 cells cultured in serum-free media for 48 h, after 5 days of plating with or without doxycycline (see schematic drawing), were placed in the lower chamber of transwell plates; parental MCF7 or MDA-MB-231 cells were seeded on the upper chamber (schematic drawing, *upper panel*). After 48 or 24 h, cells in the lower compartment were stained and fixed. Representative images with MDA-MB-231 cells are shown (*middle panels*). Migrating cells were estimated in three independent experiments (*lower panels*). Bars represent averages and standard deviations. *P < 0.05, **P < 0.01, and ***P < 0.001 using the two-sided Student’s *t* test. **b** MCF7 Tet-Off p95HER2 cells constitutively expressing a control shRNA (NT, non-targeting) or a shRNA targeting ADAM17 were co-injected orthothopically with reporter MDA-MB-231 cells expressing the luciferase gene. **c** Tumor volumes were determined at the indicated time points and expressed as average ± standard deviation. Doxycycline was withdrawn at the time indicated as 0. **d** Tumor growth determined by in vivo bioluminescence at day 16 (total photon flux emission). Results are expressed as averages and standard deviations. **e** Representative in vivo bioluminescence images of metastatic sites 20 days after tumor resection. **f** Kaplan-Meier plot showing metastasis-free survival of the same mice as in (**c**) and (**d**) after tumor resection.

Certain senescence secretomes promote cell migration [34]. Accordingly, the secretome of p95HER2-induced senescent cells promoted the migration of different breast cancer cell lines (Fig. 5a). The knockdown of ADAM17 with two independent shRNAs (Fig. 3a) abrogated the effect of the senescence secretome on cell migration (Fig. 5a and data not shown), indicating that transmembrane factors cleaved by the metalloprotease contribute to the pro-migratory effect of the senescence secretome.

The secretome of p95HER2-induced senescent cells promotes the metastatic growth of non-senescent cells [2]. Because the migratory behavior of cells in vitro (Fig. 5a) can be indicative of their metastatic potential in vivo, we analyzed the effect on ADAM17 knockdown on the non-cell autonomous prometastatic effect of p95HER2-induced senescent cells. To this end, we co-injected MCF7 Tet-Off p95HER2 cells with MDA-MB-231 cells expressing the luciferase reporter (MDA-MB-231 Luc cells) into nude mice and administered doxycycline in the drinking water to initially prevent the expression of p95HER2 from the MCF7 Tet-Off p95HER2 cells (Fig. 5b). When the tumors, populated by both cell types, reached approximately 100 mm^3^, doxycycline was removed from the drinking water to allow the expression of p95HER2. The knockdown of ADAM17 had little or no effect on the growth of MDA-MB-231 cells in the primary tumor (Fig. 5c, d). When the tumors reached approximately 300 mm^3^, we removed them and followed the fate of the remaining MDA-MB-231 Luc cells. In the majority of the cases, the primary tumors did not regrow; we only observed growth of MDA-MB-231 Luc cells in the same location of the primary tumors in two out of six mice from the control group (data not shown). Thus, MDA-MB-231 Luc cells detected in the metastatic setting left the primary tumor prior to its surgically resection. In a previous report [2], we showed that p95HER2-induced senescent cells promote the metastatic growth of MDA-MB-231 Luc cells. Consistently, we detected metastasis in the axillary ganglia and lungs of mice that had tumors composed of p95HER2-induced senescent cells and MDA-MB-231 Luc cells (Fig. 5e, f). However, the knockdown of ADAM17 prevented the metastatic growth of the reporter cells (Fig. 5e, f), arguing that factors cleaved by ADAM17 promote the metastatic growth of MDA-MB-231 Luc cells.

The results presented here show the complex regulation of ADAM17 during oncogene-induced senescence. While cellular senescence results in the accumulation of ADAM17 partially inhibited by cholesterol, the remaining activity of the metalloprotease is functionally relevant. It preferentially cleaves transmembrane molecules transcriptionally upregulated during OIS and its activity is required for some of the non-cell autonomous effects of senescent cells, including the promotion of cell migration and metastasis. These results show that ADAM17 controls the production of a subset of components of the senescence secretome, which are functionally relevant during tumor progression.

## Discussion

In this work, we show that approximately one tenth of the components of the senescence secretome are generated through protein ectodomain shedding (Additional file 1: Figure. S1c) and that one third of these are cleaved by ADAM17. This report is the first functional analysis of the contribution of ADAM17-mediated ectodomain shedding to the non-cell autonomous effects of oncogene-induced senescent cells.

The regulation of ADAM17 during senescence is complex. One of the intracellular pathways that activates ADAM17 is the MEK-ERK pathway (reviewed in [35]). Thus, one could assume that ADAM17 is activated in p95HER2-induced senescent cells, where the ERK1,2 pathway is constitutively active [15] (Figs. 2a and 4b). In fact, a recent report shows that ADAM17 is activated in Ras-induced senescent cells [14]. However, our results clearly show that activation of p95HER2 does not result in ADAM17 activation. In fact, p95HER2-induced senescent cells accumulate partially inactive ADAM17. This restriction of ADAM17 activity is likely due to the accumulation of cholesterol in senescent cells. This result contrasts with that published by Effenberger et al., who showed similar levels of ADAM17 in PC3 proliferating cells and in the same cells after induction of senescence with doxorubicin [14]. The likely explanation(s) for these apparently disparate observation may reside in differences in the cell type (PC3 and MCF7 are derived from prostate and breast cancers, respectively) or in the trigger of senescence (DNA-damage induced by Doxorubicin versus expression of p95HER2) or in both. For instance, ADAM17 levels and activity were differentially regulated in MCF7 and MCF10, which are both of breast origin and express the same oncogene.

Although the exact mechanism of ADAM17 inhibition is not known, it seems to be related to the compartmentalization of the metalloproteinase in plasma membrane subdomains where shedding substrates are not accessible [25–30]. In addition to increasing the activity of ADAM17, MβCD decreased the levels of the metalloprotease, particularly those of the processed form (Fig. 4b). A way to interpret this result is by assuming that ADAM17 inhibited by cholesterol has a longer half-life than active ADAM17. Future work will be directed to clarify whether these interpretations of the results explain not only the upregulation of the levels of ADAM17 and the restriction of its activity in p95HER2-induced senescent cells but the differences observed between p95HER2- and Ras-induced senescent cells as well.

The clear correlation between the levels of the transcripts encoding shedding substrates and the levels of ectodomains in the senescence secretome (Fig. 1d) indicates that the activity of ADAM17 is limiting in senescent cells: only substrates whose expression increases, such as Met or Areg, are cleaved. Under these limiting conditions, substrates whose expression does not increase during senescence, such as APP, are probably outcompeted and, as a result, their shedding decreases during OIS.

Despite this restriction, the remaining activity of ADAM17 clearly contributes to the protumorigenic effects of p95HER2-induced senescent cells. The data in Fig. 5a show that factors whose secretion depends on ADAM17 increase cell motility. This result, along with the fact that the effect of ADAM17 is non-cell autonomous (Fig. 5c-f), led us to conclude that the proteolytic activity of ADAM17 acts on factors that, when released, increase the metastatic ability of cancer cells.

Given the lack of modulators of the senescence secretome and the fact that the activity of ADAM17 can be upregulated by different compounds or inhibited with small-molecule synthetic inhibitors (reviewed in [36]) or monoclonal antibodies [37], our results open up a possibility that part of the effects of the senescence secretome can be modulated by regulating the activity of ADAM17. Thus, the pharmacological modulation of ADAM17 may represent a means to target the non-cell autonomous effects of cellular senescence, which may contribute to different diseases, including cancer [1].

## Conclusions

p95HER2, an oncogenic fragment of the tyrosine kinase receptor HER2, has been shown to induce senescence in a variety of breast cancer cell lines, whereas its associated secretome promotes metastasis in a non-cell autonomous manner. Analysis of this secretome showed that several soluble factors are released through ectodomain shedding, but its specific contribution has not been studied thoroughly. The present study shows that approximately 10 % of the p95HER2-induced secretome components are generated by ectodomain shedding and that the levels of shedding substrates are controlled also transcriptionally. We identified ADAM17 as the main sheddase involved in the generation of p95HER2-induced secretome. ADAM17 activity, though, is restrained by the accumulation of cellular cholesterol in senescent cells. However, the remaining activity of ADAM17 is essential to regulate the secretome composition and its functional effect. In this sense, the secretome of p95HER2-induced senescent cells that are knocked down for ADAM17 impairs migration of proliferating cells both in vivo and in vitro. Taken together, our results point out the importance of ADAM17 in the regulation of p95HER2-induced senescent secretome and its non-cell autonomous prometastatic effects.

## Additional files

Additional file 1: Figure. S1. Proteomic analysis of the secretome of p95HER2-induced senescence. **a** MCF7 Tet-Off p95HER2 cells were cultured with or without doxycycline for 1 week and stained for senescence-associated β-galactosidase. Representative images of the stained cultures are shown. **b** The secretomes of the same cells as in a were analyzed by label-free quantitative proteomics. The results are shown as unsupervised hierarchical clustering analysis corresponding to three technical replicas (a-c) of two independent experiments (1 and 2). **c** The proteins identified in b were classified according to the presence of transmembrane or glycophosphatidylinositol domains (cell membrane), signal peptide but not transmembrane domain (secreted, canonical), or the lack of these domains (secreted, unknown). See also Additional file 2: Table S1. *Doxy* doxycycline. (JPEG 585 kb) Additional file 2: Table S1. Proteins identified by label-free quantitative proteomics. *Doxy* doxycycline. (PDF 231 kb) Additional file 3: Table S2. Proteins identified by label-free quantitative proteomics secreted through the canonical pathway. *Doxy* doxycycline. (PDF 44 kb) Additional file 4: Table S3. Proteins identified by label-free quantitative proteomics with transmembrane or GPI-anchored domains and mRNA levels by transcriptomic analysis. *Doxy* doxycycline, *GPI* glycophosphatidylinositol. (PDF 52 kb) Additional file 5: Figure. S2. Alignment of peptides identified by label-free quantitative proteomics on the primary sequence of the corresponding transmembrane proteins. From Additional file 2: Table S1, we chose the type I transmembrane proteins with the largest intracellular domains and aligned the peptides identified through label-free quantitative proteomics to their primary sequence. The amino- and carboxy terminus of the proteins are marked with N and C, respectively, the signal sequence and the transmembrane domains are represented by *red* boxes, and peptides identified by mass spectrometry are represented by *yellow* boxes. The numbers on top of the schematics represent amino acid positions. In each case, the number of amino acids corresponding to the peptides identified by mass spectrometry and the number of amino acids of the extracellular and intracellular domains, as well as the percentages, are shown. (JPEG 265 kb) Additional file 6: Table S4. List of confirmed proteins from label-free proteomic analysis. (PDF 36 kb) Additional file 7: Table S5. Proteins identified by label-free quantitative proteomics in MCF7 Tet-Off p95HER2 shNT and shADAM17. *Doxy* doxycycline, *GPI* glycophosphatidylinositol. (PDF 44 kb) Additional file 8: Figure. S3.
**a** Short-term cholesterol depletion activates the shedding of AP-tagged TGF-α and Areg. MCF7 cells expressing AP-tagged TGF-α or Areg were cultured with or without doxycycline and treated with MβCD or vehicle for 1 h as indicated. AP was quantified in serum-free conditioned media and cell lysates. Data shown represent the averages and standard deviations of three independent experiments. ***P* < 0.01 using the two-sided Student’s *t* test. **b** The secretome of p95HER2-induced senescent cells contains factors that activate the EGFR. A431 cells were stimulated with conditioned media obtained from culturing MCF7 Tet-Off p95HER2 cells shNT or shADAM17 in serum-free media for 48 h, after 5 days of plating with or without doxycycline (see schematic drawing). Then A431 cell lysates were analyzed by Western blot by using the indicated antibodies. Quantification of densitometric data is shown. *AP* alkaline phosphatase, *Areg* amphiregulin, *Doxy* doxycycline, *MβCD* methyl-beta-cyclodextrin, *TGF-α* transforming growth factor-alpha. (JPEG 275 kb)

## Abbreviations

AP: Alkaline phosphatase
AP-Areg: Alkaline phosphatase-tagged amphiregulin
AP-BTC: Alkaline phosphatase-tagged betacellulin
APP: Amyloid precursor protein
AP-TGF-α: Alkaline phosphatase-tagged transforming growth factor-alpha
Areg: Amphiregulin
EGF: Epidermal growth factor
EGFR: Epidermal growth factor receptor
FBS: Fetal bovine serum
MβCD: Methyl-beta-cyclodextrin
OIS: Oncogene-induced senescence
PBS: Phosphate-buffered saline
