# Supplementary figures and images for: Role of ADAM17 in the non-cell autonomous effects of oncogene-induced senescence
Source: Breast Cancer Res. 2015 Aug 12;17(1):106. doi: 10.1186/s13058-015-0619-7 (PMC4532141; doi:10.1186/s13058-015-0619-7)

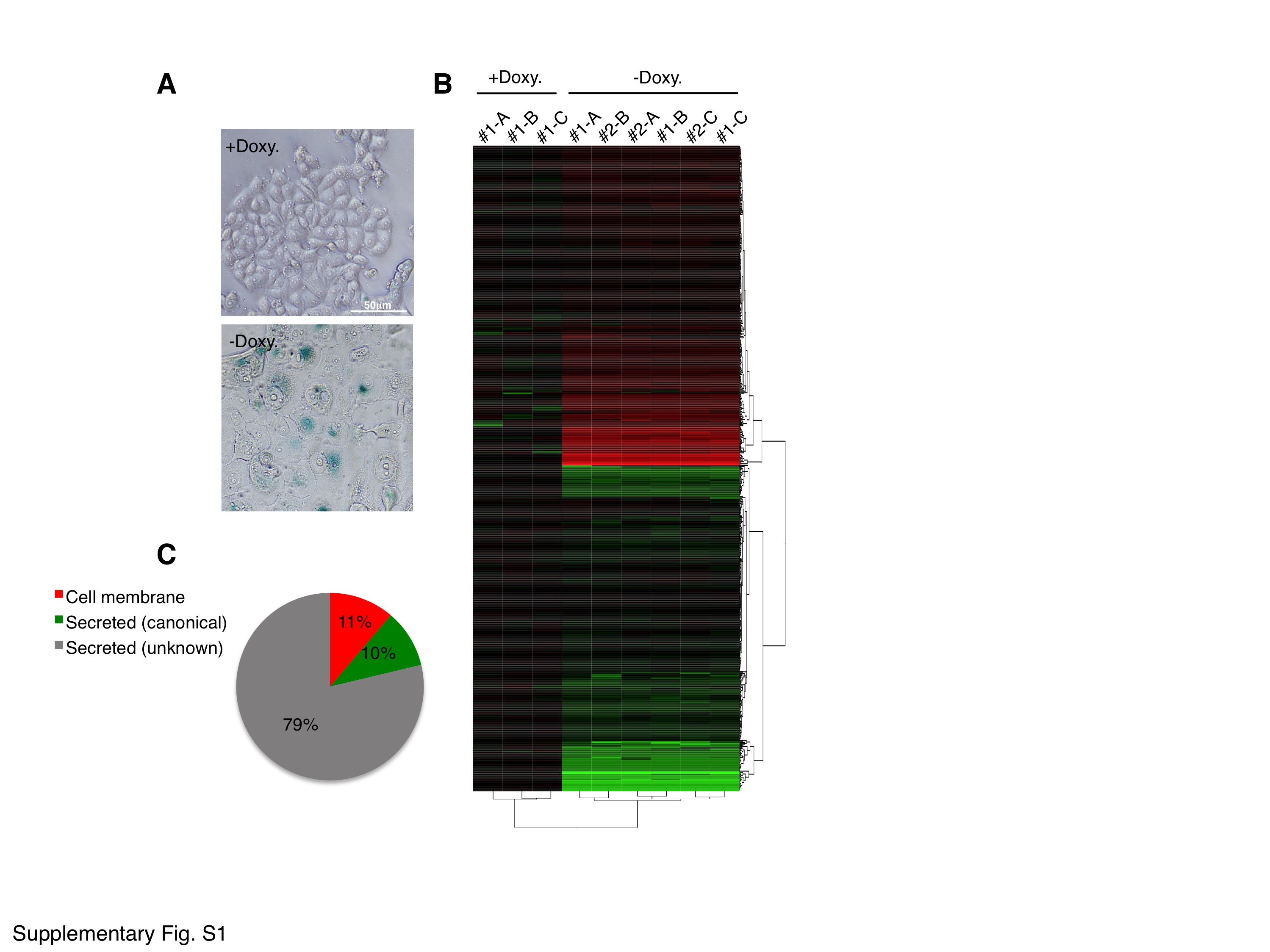

Supplement: Additional file 1: Figure. S1. — Proteomic analysis of the secretome of p95HER2-induced senescence. a MCF7 Tet-Off p95HER2 cells were cultured with or without doxycycline for 1 week and stained for senescence-associated β-galactosidase. Representative images of the stained cultures are shown. b The secretomes of the same cells as in a were analyzed by label-free quantitative proteomics. The results are shown as unsupervised hierarchical clustering analysis corresponding to three technical replicas (a-c) of two independent experiments (1 and 2). c The proteins identified in b were classified according to the presence of transmembrane or glycophosphatidylinositol domains (cell membrane), signal peptide but not transmembrane domain (secreted, canonical), or the lack of these domains (secreted, unknown). See also Additional file 2: Table S1. Doxy doxycycline. (JPEG 585 kb) [file 13058_2015_619_MOESM1_ESM.jpg]

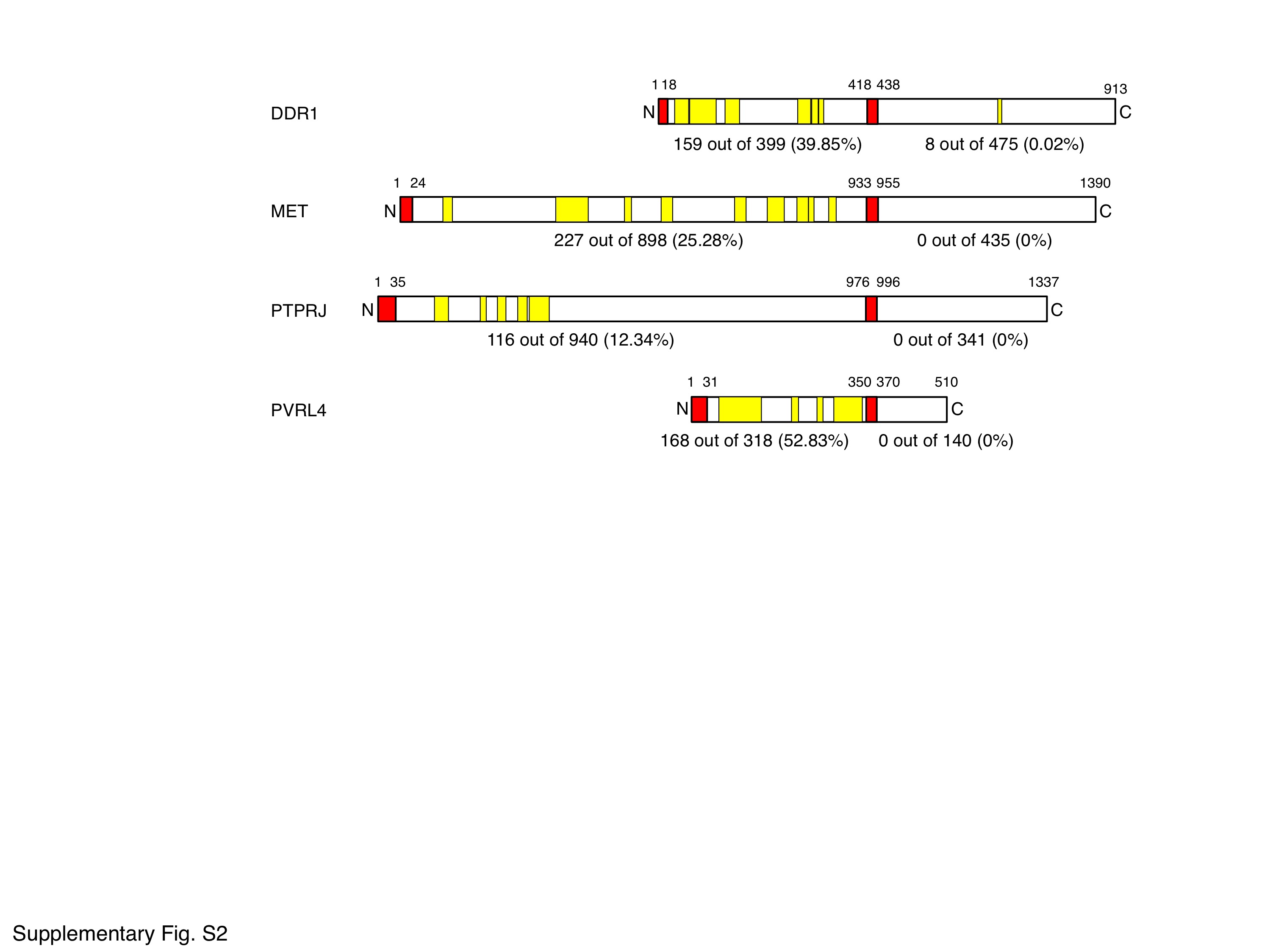

Supplement: Additional file 5: Figure. S2. — Alignment of peptides identified by label-free quantitative proteomics on the primary sequence of the corresponding transmembrane proteins. From Additional file 2: Table S1, we chose the type I transmembrane proteins with the largest intracellular domains and aligned the peptides identified through label-free quantitative proteomics to their primary sequence. The amino- and carboxy terminus of the proteins are marked with N and C, respectively, the signal sequence and the transmembrane domains are represented by red boxes, and peptides identified by mass spectrometry are represented by yellow boxes. The numbers on top of the schematics represent amino acid positions. In each case, the number of amino acids corresponding to the peptides identified by mass spectrometry and the number of amino acids of the extracellular and intracellular domains, as well as the percentages, are shown. (JPEG 265 kb) [file 13058_2015_619_MOESM5_ESM.jpg]

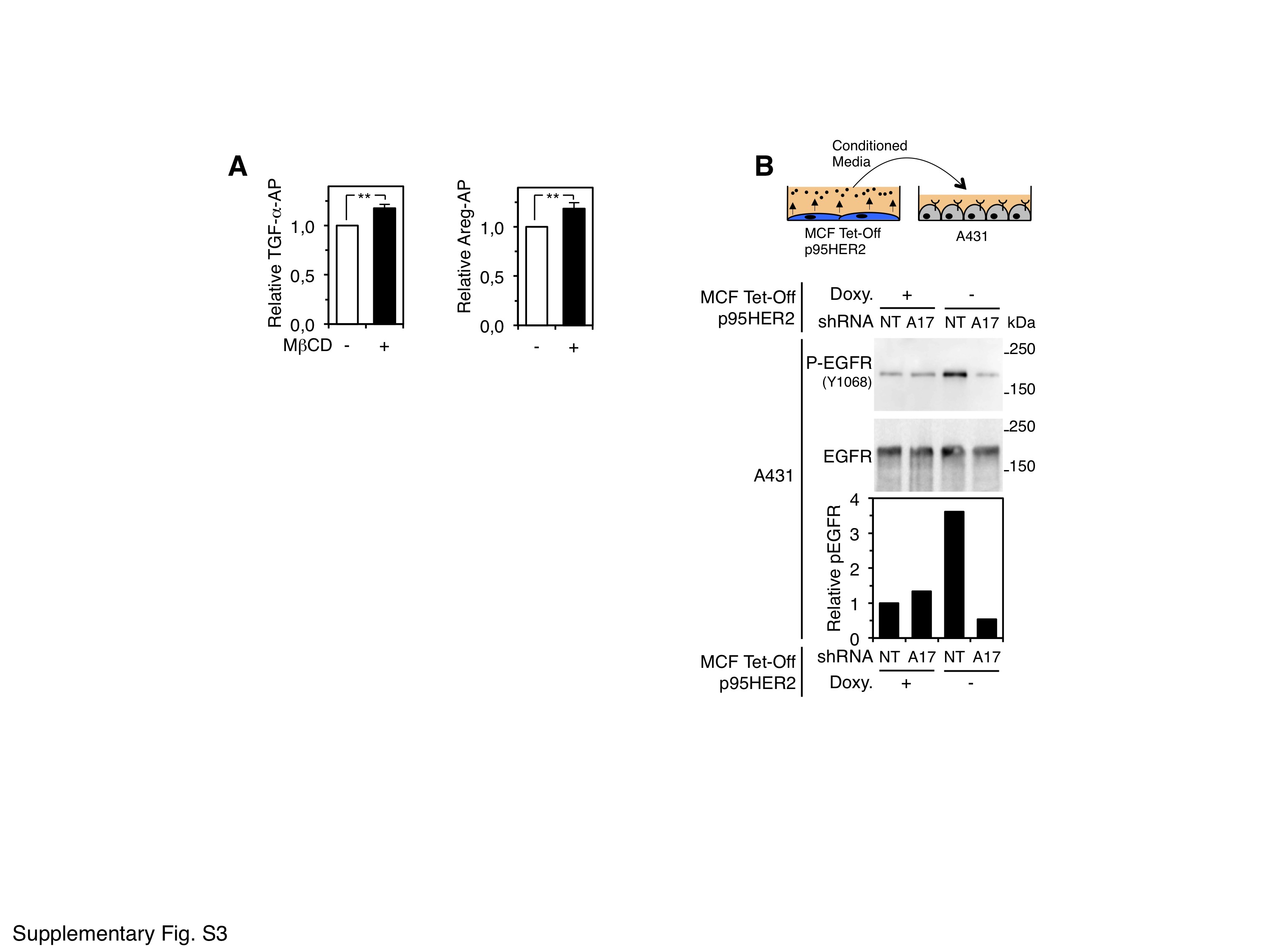

Supplement: Additional file 8: Figure. S3. — a Short-term cholesterol depletion activates the shedding of AP-tagged TGF-α and Areg. MCF7 cells expressing AP-tagged TGF-α or Areg were cultured with or without doxycycline and treated with MβCD or vehicle for 1 h as indicated. AP was quantified in serum-free conditioned media and cell lysates. Data shown represent the averages and standard deviations of three independent experiments. **P < 0.01 using the two-sided Student’s t test. b The secretome of p95HER2-induced senescent cells contains factors that activate the EGFR. A431 cells were stimulated with conditioned media obtained from culturing MCF7 Tet-Off p95HER2 cells shNT or shADAM17 in serum-free media for 48 h, after 5 days of plating with or without doxycycline (see schematic drawing). Then A431 cell lysates were analyzed by Western blot by using the indicated antibodies. Quantification of densitometric data is shown. AP alkaline phosphatase, Areg amphiregulin, Doxy doxycycline, MβCD methyl-beta-cyclodextrin, TGF-α transforming growth factor-alpha. (JPEG 275 kb) [file 13058_2015_619_MOESM8_ESM.jpg]
