# Supplementary material for: Role of ADAM17 in the non-cell autonomous effects of oncogene-induced senescence
Source: Breast Cancer Res. 2015 Aug 12;17(1):106. doi: 10.1186/s13058-015-0619-7 (PMC4532141; doi:10.1186/s13058-015-0619-7)
Supplement: Additional file 2: Table S1. — Proteins identified by label-free quantitative proteomics. Doxy doxycycline. (PDF 231 kb) [file 13058_2015_619_MOESM2_ESM.pdf]

Supplementary Table SI. Proteins identified by label-free quantitative proteomics

	Proteins identified in the extracelular media (Spectral Counts)								
	+Doxy.			-Doxy.					
	MCF7 Tet-Off p95HER2 #1			MCF7 Tet-Off p95HER2 #1			MCF7 Tet-Off p95HER2 #2		
Gene Name	A	B	C	A	B	C	A	B	C
FLNA	261,53	256,69	250,13	259,58	268,58	275,56	245,17	247,51	261,77
AGRN	157,12	171,83	169,80	22,22	28,67	27,17	26,27	26,78	29,42
ACTN4	132,26	135,77	137,26	127,12	133,81	133,90	132,32	134,84	136,97
ACTG1	151,15	144,26	147,43	199,13	200,72	190,17	204,31	190,25	162,34
KRT8	107,40	106,07	107,78	105,79	112,79	114,49	107,99	100,67	103,49
FLNB	71,60	72,13	77,28	75,56	86,02	89,27	72,00	82,20	81,17
TUBB	91,49	96,52	96,59	94,23	98,45	99,94	86,59	89,59	87,26
TUBB4B	85,52	90,16	90,49	90,68	100,36	97,03	87,56	91,43	90,30
HSPA8	76,57	79,55	84,39	81,79	88,89	87,33	91,45	91,43	88,27
ACTN1	79,55	81,67	79,31	123,57	130,95	134,87	142,05	139,46	134,94
KRT19	68,62	73,19	67,11	75,56	65,00	64,04	60,32	59,11	58,85
THBS1	101,43	98,65	87,44	33,78	35,37	38,81	27,24	34,17	33,48
IGFBP5	49,72	54,10	61,01	0,00	0,00	0,00	0,00	0,00	0,00
FASN	69,61	65,76	72,19	86,23	86,98	82,47	68,10	67,42	62,91
ENO1	83,53	84,86	79,31	72,01	65,95	67,92	65,19	67,42	70,01
ALDOA	69,61	56,22	62,02	81,79	78,38	84,41	80,75	87,74	89,29
KRT18	60,66	55,16	54,91	78,23	70,73	72,77	55,46	62,80	61,89
CFL1	70,60	83,80	79,31	40,00	46,84	42,69	42,81	47,10	38,56
HSPB1	61,65	58,34	63,04	49,78	46,84	44,63	53,51	48,95	49,72
YWHAZ	64,64	64,70	73,21	59,56	60,22	71,80	63,24	66,50	78,13
PKM2	57,68	56,22	55,92	66,67	65,95	66,95	64,21	64,65	64,94
EEF2	65,63	70,01	57,96	112,01	112,79	111,58	92,43	90,51	83,20

HSP90AA1	68,62	62,58	68,12	86,23	86,98	91,21	93,40	92,36	90,30
IGFBP2	47,73	51,98	58,97	17,78	19,12	17,47	17,51	16,62	21,31
HSP90AB1	60,66	66,82	65,07	71,12	77,42	77,62	76,86	78,50	76,10
HSPA1A	53,70	48,79	51,86	46,23	46,84	45,60	51,56	45,25	44,64
IQGAP1	39,78	36,06	38,64	27,56	27,72	27,17	28,22	24,94	27,40
AHNAK	41,77	35,00	42,71	42,67	36,32	38,81	39,89	35,10	39,57
QSOX1	33,81	32,88	29,49	60,45	57,35	57,25	54,48	54,49	55,80
BASP1	44,75	48,79	40,67	40,00	43,01	41,72	41,84	36,02	41,60
PTPRF	42,76	56,22	49,82	0,00	0,00	0,00	0,00	0,00	0,00
CLSTN1	43,75	39,25	37,62	14,22	14,34	16,50	14,59	16,62	16,23
STC2	39,78	42,43	44,74	8,00	7,65	4,85	6,81	6,46	5,07
PXDN	27,84	31,82	27,45	4,44	2,87	1,94	5,84	3,69	3,04
CLU	29,83	30,76	26,44	7,11	10,51	7,76	6,81	6,46	5,07
TUBB3	42,76	45,61	45,76	54,23	62,13	60,16	59,35	56,34	53,78
TUBA1B	46,74	41,37	43,72	54,23	53,53	54,34	48,65	48,95	45,66
PLEC	20,88	16,97	25,42	44,45	46,84	50,45	37,94	34,17	34,50
VCP	45,74	45,61	45,76	55,12	51,61	56,28	49,62	61,88	54,79
TRIM28	38,78	46,67	46,77	27,56	33,45	38,81	29,19	40,64	34,50
TUBA1C	42,76	41,37	42,71	52,45	50,66	53,37	47,67	48,03	44,64
HNRNPA2B1	35,80	38,19	34,57	28,45	28,67	26,20	32,11	26,78	28,41
PPIA	50,72	45,61	44,74	32,89	34,41	37,84	37,94	41,56	38,56
GPI	50,72	40,31	43,72	33,78	25,81	23,29	29,19	27,71	26,38
RDX	22,87	26,52	32,54	20,45	16,25	15,52	20,43	20,32	16,23
GDI2	42,76	39,25	35,59	35,56	29,63	34,93	38,92	31,40	39,57
UGDH	49,72	48,79	42,71	28,45	26,76	27,17	18,49	23,09	23,34
TPI1	37,79	41,37	43,72	37,34	36,32	35,90	35,03	37,87	43,63
TFF1	31,82	37,13	37,62	10,67	11,47	12,61	9,73	12,01	13,19
LMNA	30,83	32,88	29,49	43,56	47,79	43,66	39,89	38,79	36,53

TPM4	35,80	37,13	37,62	40,89	43,01	41,72	38,92	42,48	39,57
SEMA3C	25,86	22,28	23,39	0,89	0,96	0,00	0,97	0,00	0,00
LTBP1	11,93	12,73	14,24	0,00	0,00	0,00	0,00	0,00	0,00
UBA1	41,77	41,37	45,76	31,11	34,41	33,96	34,05	35,10	35,51
G6PD	38,78	38,19	34,57	32,89	37,28	38,81	36,00	30,48	33,48
SPTAN1	16,91	14,85	17,29	42,67	38,23	28,14	33,08	31,40	28,41
EEF1A1	33,81	29,70	38,64	40,89	38,23	42,69	36,97	41,56	38,56
YWHAE	32,82	30,76	32,54	22,22	27,72	27,17	28,22	30,48	32,47
ATIC	29,83	30,76	23,39	23,11	19,12	21,35	23,35	19,40	23,34
ALCAM	26,85	27,58	23,39	6,22	7,65	9,70	3,89	6,46	7,10
CST3	24,86	21,21	20,34	3,56	5,73	3,88	4,86	4,62	5,07
YWHAB	30,83	32,88	35,59	28,45	31,54	33,96	31,13	36,02	35,51
NRCAM	18,89	16,97	14,24	0,00	0,00	0,00	0,00	0,00	0,00
DPP3	31,82	28,64	32,54	16,00	14,34	12,61	13,62	15,70	11,16
PRDX1	29,83	31,82	27,45	23,11	24,85	22,32	23,35	25,86	28,41
NME2	30,83	25,46	26,44	25,78	28,67	28,14	28,22	27,71	23,34
STIP1	28,84	22,28	25,42	26,67	30,59	32,02	34,05	32,33	32,47
CPE	27,84	28,64	24,40	11,56	12,43	7,76	9,73	11,08	9,13
PEBP1	25,86	20,15	27,45	16,00	16,25	19,41	16,54	19,40	15,22
CELSR2	22,87	25,46	23,39	0,00	0,00	0,00	0,00	0,00	0,00
APP	26,85	26,52	31,52	14,22	11,47	11,64	13,62	12,01	12,18
MARCKS	30,83	31,82	32,54	14,22	13,38	14,55	15,57	14,78	17,25
HNRNPK	26,85	27,58	25,42	21,34	19,12	19,41	21,40	18,47	18,26
IGFBP4	23,87	24,40	19,32	7,11	5,73	6,79	5,84	5,54	4,06
NME1	22,87	21,21	21,35	22,22	20,07	22,32	22,38	24,01	24,35
TKT	24,86	26,52	26,44	24,89	21,03	20,38	17,51	21,24	19,28
PFN1	24,86	32,88	29,49	26,67	31,54	29,11	28,22	29,55	31,45
ACTR3	28,84	25,46	26,44	27,56	28,67	31,05	32,11	28,63	31,45

P4HB	20,88	15,91	18,30	63,12	64,04	64,04	62,27	54,49	53,78
UBA52	25,86	30,76	29,49	24,00	24,85	24,26	28,22	31,40	26,38
EEF1A2	24,86	24,40	25,42	35,56	30,59	35,90	28,22	30,48	30,44
CALR	19,89	18,03	12,20	46,23	49,70	46,57	38,92	43,41	43,63
YWHAQ	26,85	26,52	27,45	24,00	26,76	26,20	24,32	31,40	26,38
HMGB1	31,82	36,06	28,47	24,89	24,85	21,35	20,43	28,63	19,28
PRDX6	27,84	22,28	20,34	22,22	20,07	22,32	25,30	25,86	25,37
SFN	20,88	22,28	27,45	24,89	30,59	32,99	31,13	32,33	34,50
FBP1	15,91	16,97	20,34	1,78	0,00	1,94	0,97	2,77	1,01
CTTN	18,89	20,15	20,34	14,22	13,38	17,47	18,49	16,62	18,26
YBX1	22,87	23,34	22,37	27,56	25,81	28,14	23,35	28,63	30,44
CLTC	20,88	16,97	20,34	81,79	72,64	69,86	62,27	66,50	63,92
LDHA	22,87	22,28	16,27	31,11	24,85	25,23	25,30	25,86	22,32
HSPA5	19,89	20,15	22,37	62,23	63,08	56,28	64,21	61,88	58,85
COL12A1	15,91	19,09	21,35	0,00	0,00	0,00	0,00	0,00	0,00
CORO1B	19,89	20,15	23,39	6,22	6,69	8,73	11,68	7,39	7,10
SLC9A3R1	22,87	24,40	21,35	12,45	11,47	13,58	12,65	15,70	14,21
PGK1	23,87	23,34	21,35	28,45	30,59	30,08	34,05	32,33	38,56
LSR	15,91	16,97	17,29	11,56	13,38	12,61	14,59	12,01	14,21
CDH1	20,88	20,15	21,35	8,89	10,51	11,64	8,76	10,16	9,13
TAGLN2	28,84	26,52	32,54	37,34	34,41	35,90	37,94	36,94	36,53
EFEMP1	17,90	16,97	17,29	0,00	0,00	0,00	0,00	0,00	0,00
NPEPPS	14,92	14,85	14,24	18,67	15,29	17,47	17,51	20,32	19,28
HDGF	19,89	21,21	19,32	16,89	14,34	17,47	20,43	18,47	15,22
ST14	9,94	8,49	13,22	4,44	3,82	6,79	3,89	4,62	4,06
HSPA4	25,86	27,58	28,47	29,34	35,37	35,90	36,00	34,17	36,53
GDI1	23,87	24,40	22,37	24,89	23,90	27,17	24,32	24,01	32,47
PTPRK	15,91	15,91	18,30	1,78	0,96	0,97	0,97	1,85	1,01

S100A11	31,82	32,88	29,49	54,23	56,39	49,48	39,89	42,48	33,48
SH3BGRL	23,87	23,34	27,45	6,22	8,60	4,85	6,81	4,62	6,09
GSN	21,88	24,40	28,47	16,89	18,16	20,38	19,46	19,40	22,32
PRDX2	16,91	20,15	16,27	14,22	13,38	11,64	17,51	12,93	13,19
TIMP1	16,91	14,85	18,30	15,11	16,25	14,55	13,62	14,78	13,19
YWHAG	20,88	21,21	25,42	20,45	25,81	25,23	24,32	24,01	28,41
CTSD	23,87	21,21	21,35	12,45	13,38	13,58	18,49	15,70	18,26
EZR	24,86	18,03	22,37	24,89	25,81	23,29	19,46	24,01	21,31
PPA1	15,91	18,03	14,24	21,34	21,03	19,41	23,35	22,17	22,32
SULF2	19,89	20,15	18,30	0,00	0,00	0,00	0,00	0,00	0,00
NRP1	8,95	7,42	9,15	3,56	0,96	2,91	0,97	1,85	2,03
GSTM3	14,92	14,85	15,25	8,00	8,60	8,73	9,73	8,31	5,07
EEF1G	20,88	21,21	19,32	37,34	34,41	29,11	30,16	31,40	32,47
DKK1	16,91	18,03	13,22	1,78	0,96	0,97	0,97	0,92	1,01
FREM2	0,99	4,24	3,05	0,00	0,00	0,00	0,00	0,00	0,00
TPT1	18,89	19,09	19,32	33,78	46,84	41,72	38,92	53,57	32,47
CTNNA1	14,92	15,91	15,25	2,67	4,78	1,94	1,95	2,77	3,04
BCAM	14,92	15,91	14,24	0,00	0,00	0,00	0,00	0,00	0,00
SPINT1	15,91	14,85	12,20	10,67	13,38	8,73	11,68	14,78	12,18
NPM1	33,81	36,06	32,54	38,23	43,01	42,69	39,89	41,56	42,61
ACLY	14,92	15,91	12,20	19,56	25,81	25,23	25,30	19,40	21,31
PRKCSH	12,93	10,61	12,20	24,89	23,90	23,29	27,24	24,94	28,41
NUCB1	8,95	8,49	9,15	1,78	1,91	0,97	1,95	1,85	2,03
APLP2	10,94	7,42	7,12	6,22	6,69	4,85	5,84	2,77	6,09
GLG1	5,97	7,42	7,12	1,78	1,91	0,97	1,95	2,77	2,03
SYNCRIP	15,91	15,91	16,27	13,34	17,21	18,44	17,51	16,62	19,28
PSME1	19,89	22,28	23,39	12,45	12,43	10,67	13,62	13,85	10,15
ALB	11,93	13,79	14,24	8,00	13,38	7,76	10,70	10,16	9,13

LYPD3	14,92	14,85	15,25	6,22	6,69	5,82	5,84	5,54	7,10
AMOTL1	1,99	2,12	2,03	0,00	0,00	0,00	0,00	0,00	0,00
PGD	17,90	20,15	20,34	13,34	15,29	13,58	19,46	17,55	17,25
NEO1	10,94	15,91	12,20	0,00	0,00	0,00	0,00	0,00	0,00
TPM3	16,91	16,97	17,29	16,89	14,34	15,52	16,54	15,70	14,21
LXN	12,93	14,85	16,27	7,11	6,69	6,79	7,78	7,39	7,10
CALM1	15,91	14,85	17,29	9,78	9,56	7,76	6,81	7,39	11,16
CLIC1	13,92	14,85	17,29	19,56	21,03	21,35	25,30	22,17	22,32
AARS	16,91	15,91	12,20	7,11	6,69	8,73	7,78	7,39	8,12
GFRA1	5,97	5,30	6,10	0,00	0,00	0,00	0,00	0,00	0,00
TFF3	16,91	15,91	17,29	0,00	0,00	0,00	0,00	0,00	0,00
TPM1	15,91	16,97	18,30	16,89	18,16	17,47	18,49	19,40	16,23
PARK7	20,88	16,97	18,30	12,45	16,25	13,58	15,57	14,78	13,19
ALDOC	13,92	11,67	13,22	15,11	14,34	14,55	16,54	16,62	19,28
HNRNPA1	14,92	10,61	11,19	7,11	7,65	2,91	7,78	5,54	6,09
SFPQ	7,96	12,73	7,12	5,33	3,82	5,82	3,89	4,62	4,06
TUBB6	21,88	24,40	23,39	24,00	29,63	25,23	26,27	26,78	24,35
CA2	21,88	21,21	17,29	1,78	1,91	0,97	5,84	1,85	4,06
LMNB1	15,91	16,97	12,20	3,56	1,91	0,00	0,00	0,92	0,00
EPS15L1	9,94	7,42	12,20	0,00	0,00	0,00	0,00	0,00	0,00
B2M	13,92	10,61	13,22	12,45	12,43	10,67	11,68	8,31	11,16
VCL	11,93	13,79	17,29	28,45	32,50	32,99	40,86	37,87	40,59
KRT1	14,92	16,97	13,22	16,89	12,43	17,47	27,24	24,94	21,31
CAP1	14,92	13,79	16,27	21,34	21,98	23,29	21,40	20,32	20,29
XRCC5	10,94	7,42	11,19	6,22	8,60	10,67	6,81	10,16	6,09
BLVRB	12,93	14,85	15,25	21,34	23,90	18,44	22,38	22,17	23,34
CBX3	12,93	16,97	15,25	13,34	13,38	12,61	13,62	12,93	14,21
PGAM1	12,93	13,79	13,22	17,78	19,12	18,44	20,43	23,09	23,34

PABPC1	10,94	9,55	13,22	14,22	17,21	14,55	13,62	13,85	16,23
SDF4	11,93	11,67	13,22	7,11	8,60	6,79	4,86	7,39	6,09
GGH	13,92	11,67	11,19	0,00	0,00	0,00	0,97	0,00	1,01
HSPH1	13,92	14,85	13,22	32,00	32,50	30,08	37,94	37,87	33,48
SOD1	13,92	12,73	14,24	9,78	12,43	7,76	8,76	8,31	5,07
FUBP1	13,92	9,55	10,17	13,34	8,60	9,70	10,70	12,01	12,18
PTBP1	12,93	13,79	17,29	12,45	9,56	14,55	14,59	11,08	10,15
PSAP	11,93	11,67	14,24	40,00	39,19	40,75	44,75	39,71	41,60
PSMA5	8,95	9,55	11,19	11,56	11,47	9,70	11,68	11,08	15,22
MYL6	9,94	10,61	13,22	10,67	8,60	10,67	6,81	7,39	9,13
EEF1B2	12,93	13,79	14,24	18,67	25,81	20,38	21,40	23,09	20,29
TPD52L2	9,94	10,61	10,17	15,11	18,16	19,41	14,59	18,47	20,29
PCBP1	9,94	7,42	10,17	8,89	9,56	5,82	9,73	10,16	10,15
HNRNPD	9,94	11,67	11,19	10,67	9,56	7,76	9,73	9,24	8,12
GPC1	5,97	9,55	8,13	0,00	0,00	0,00	0,00	0,00	0,00
DAG1	9,94	11,67	11,19	2,67	1,91	2,91	1,95	1,85	3,04
YWHAH	10,94	13,79	14,24	8,00	12,43	12,61	11,68	12,93	12,18
LASP1	13,92	14,85	11,19	16,00	15,29	16,50	13,62	17,55	16,23
HNRNPL	10,94	12,73	14,24	8,00	8,60	7,76	6,81	6,46	9,13
RAD23B	17,90	11,67	11,19	13,34	13,38	13,58	11,68	13,85	9,13
BAG3	6,96	5,30	5,08	16,89	12,43	14,55	11,68	16,62	13,19
VASP	13,92	14,85	8,13	12,45	11,47	8,73	12,65	8,31	12,18
MDH1	16,91	12,73	14,24	8,00	11,47	6,79	9,73	11,08	9,13
RPLP2	12,93	12,73	13,22	16,00	15,29	18,44	15,57	13,85	17,25
KPNB1	17,90	18,03	19,32	21,34	20,07	21,35	20,43	24,01	25,37
SDC4	11,93	13,79	13,22	8,89	10,51	9,70	11,68	10,16	11,16
MARCKSL1	12,93	14,85	17,29	5,33	4,78	4,85	5,84	5,54	7,10
LAP3	10,94	11,67	9,15	8,00	6,69	8,73	9,73	6,46	9,13

GDF15	14,92	15,91	18,30	15,11	16,25	15,52	16,54	16,62	17,25
MDK	12,93	12,73	12,20	4,44	5,73	4,85	3,89	5,54	2,03
KRT10	9,94	7,42	7,12	8,89	6,69	9,70	14,59	13,85	13,19
GLO1	8,95	8,49	11,19	9,78	7,65	6,79	7,78	8,31	8,12
HMGB2	13,92	14,85	13,22	2,67	0,96	0,97	1,95	2,77	0,00
EEF1D	9,94	12,73	13,22	16,89	18,16	18,44	19,46	19,40	19,28
PSMA4	11,93	10,61	12,20	8,00	7,65	7,76	10,70	12,01	12,18
RNH1	10,94	11,67	12,20	13,34	13,38	12,61	11,68	13,85	9,13
TARS	9,94	9,55	10,17	13,34	13,38	11,64	13,62	12,93	15,22
ENO2	13,92	15,91	13,22	0,00	7,65	10,67	11,68	12,93	12,18
GAA	8,95	7,42	11,19	1,78	0,96	0,97	1,95	1,85	1,01
DDR1	5,97	5,30	5,08	0,00	0,00	0,00	0,00	0,92	0,00
HNRNPH1	6,96	10,61	9,15	7,11	9,56	6,79	7,78	7,39	5,07
HNRNPAB	9,94	11,67	11,19	8,89	6,69	7,76	9,73	10,16	10,15
CAPZB	13,92	12,73	13,22	9,78	9,56	6,79	10,70	11,08	11,16
SPTBN1	5,97	3,18	3,05	24,89	25,81	22,32	25,30	22,17	25,37
LAMA5	19,89	27,58	22,37	3,56	0,00	4,85	0,00	0,92	1,01
RBMX	9,94	8,49	10,17	7,11	5,73	5,82	5,84	6,46	6,09
SET	14,92	15,91	15,25	16,89	16,25	21,35	17,51	15,70	14,21
DBNL	7,96	9,55	12,20	4,44	3,82	3,88	3,89	5,54	7,10
PSMA1	13,92	13,79	9,15	10,67	12,43	9,70	9,73	11,08	14,21
FUS	10,94	8,49	10,17	10,67	6,69	9,70	5,84	7,39	7,10
PA2G4	8,95	9,55	13,22	9,78	11,47	13,58	10,70	13,85	15,22
CDC37	8,95	9,55	10,17	15,11	20,07	16,50	18,49	18,47	18,26
NUCB2	6,96	6,36	4,07	4,44	4,78	3,88	3,89	4,62	6,09
SLC3A2	9,94	11,67	9,15	11,56	12,43	11,64	13,62	12,01	10,15
CGREF1	5,97	5,30	6,10	0,00	0,00	0,00	0,00	0,00	0,00
PLOD3	9,94	10,61	9,15	25,78	21,98	24,26	29,19	31,40	34,50

CAPZA1	12,93	12,73	9,15	8,00	9,56	11,64	12,65	10,16	12,18
GOT1	13,92	11,67	11,19	7,11	9,56	8,73	9,73	8,31	11,16
OTUB1	8,95	8,49	7,12	6,22	7,65	6,79	6,81	8,31	7,10
HNRNPF	8,95	10,61	8,13	5,33	7,65	4,85	6,81	6,46	4,06
KRT9	8,95	10,61	9,15	7,11	8,60	7,76	11,68	17,55	12,18
PRDX5	8,95	9,55	13,22	14,22	12,43	13,58	16,54	13,85	15,22
RNPEP	9,94	5,30	13,22	4,44	5,73	5,82	2,92	3,69	5,07
TXN	11,93	13,79	12,20	9,78	12,43	9,70	8,76	11,08	11,16
HSPG2	7,96	8,49	5,08	0,89	0,00	0,00	0,97	0,92	1,01
GAPDH	8,95	12,73	13,22	16,00	14,34	13,58	13,62	15,70	19,28
CSRP1	7,96	7,42	8,13	9,78	9,56	9,70	12,65	12,93	12,18
EPHB4	8,95	11,67	8,13	0,00	0,00	0,00	0,00	0,00	0,00
HMGB3	4,97	9,55	10,17	2,67	2,87	3,88	3,89	3,69	7,10
LGALS3	9,94	12,73	15,25	5,33	5,73	3,88	4,86	3,69	5,07
GRN	6,96	9,55	7,12	14,22	11,47	9,70	13,62	12,93	11,16
FKBP4	11,93	12,73	12,20	11,56	9,56	11,64	12,65	11,08	15,22
CSDA	7,96	8,49	9,15	9,78	9,56	6,79	8,76	10,16	9,13
RPL5	9,94	12,73	10,17	7,11	8,60	8,73	11,68	13,85	10,15
PDAP1	9,94	9,55	11,19	8,89	8,60	6,79	10,70	6,46	5,07
TPD52	7,96	8,49	6,10	8,89	4,78	10,67	9,73	7,39	9,13
CSE1L	13,92	11,67	10,17	6,22	4,78	7,76	5,84	9,24	9,13
DNPEP	9,94	10,61	12,20	4,44	2,87	2,91	1,95	3,69	4,06
PSMB1	11,93	10,61	11,19	10,67	8,60	6,79	7,78	5,54	5,07
MIF	9,94	9,55	8,13	7,11	8,60	6,79	7,78	9,24	9,13
SND1	6,96	4,24	9,15	13,34	12,43	11,64	9,73	11,08	10,15
SERBP1	11,93	7,42	8,13	11,56	8,60	11,64	9,73	9,24	8,12
FAM49B	7,96	10,61	13,22	5,33	6,69	7,76	7,78	9,24	9,13
EPPK1	2,98	3,18	6,10	24,89	24,85	26,20	21,40	24,01	23,34

PNP	5,97	8,49	6,10	23,11	21,98	22,32	26,27	24,01	21,31
HN1	7,96	8,49	5,08	8,89	10,51	8,73	7,78	8,31	8,12
HNRPDL	8,95	7,42	8,13	0,00	0,00	5,82	0,00	0,00	7,10
SF3B3	7,96	6,36	7,12	0,89	0,96	0,97	1,95	2,77	2,03
KRT2	0,00	8,49	7,12	8,00	7,65	8,73	8,76	9,24	6,09
UBE2L3	10,94	8,49	7,12	8,00	8,60	6,79	9,73	9,24	6,09
PDIA3	5,97	8,49	3,05	24,00	26,76	25,23	20,43	24,94	24,35
EIF5A	14,92	10,61	9,15	11,56	11,47	10,67	10,70	12,93	12,18
CADM1	3,98	4,24	4,07	0,00	0,00	0,00	0,00	0,00	0,00
HMGN1	8,95	11,67	7,12	4,44	2,87	3,88	3,89	3,69	5,07
PCBP2	9,94	8,49	9,15	5,33	5,73	6,79	3,89	6,46	8,12
CCT8	7,96	10,61	9,15	14,22	7,65	10,67	12,65	12,93	12,18
MDH2	5,97	7,42	6,10	5,33	5,73	5,82	4,86	5,54	7,10
MYL12A	6,96	7,42	7,12	8,00	9,56	8,73	10,70	7,39	9,13
RPL12	6,96	8,49	8,13	8,89	9,56	8,73	9,73	9,24	11,16
PPP2R1A	10,94	13,79	13,22	16,00	15,29	13,58	18,49	22,17	16,23
ARPC3	8,95	10,61	8,13	9,78	9,56	8,73	10,70	11,08	9,13
PDIA6	2,98	1,06	3,05	19,56	21,98	27,17	19,46	21,24	20,29
ILF3	8,95	10,61	6,10	4,44	4,78	4,85	3,89	6,46	5,07
CUTA	6,96	9,55	7,12	4,44	3,82	2,91	1,95	2,77	2,03
PPP1CA	5,97	8,49	5,08	4,44	4,78	5,82	7,78	5,54	4,06
UBE2N	8,95	8,49	8,13	6,22	7,65	7,76	5,84	7,39	7,10
PREX1	1,99	2,12	2,03	0,00	0,00	0,00	0,00	0,00	0,00
DNASE2	5,97	6,36	5,08	7,11	5,73	4,85	6,81	7,39	5,07
HNRNPU	9,94	7,42	9,15	7,11	3,82	6,79	4,86	4,62	7,10
BTD	4,97	4,24	3,05	0,00	0,00	0,00	0,00	0,00	0,00
COL18A1	4,97	4,24	5,08	0,00	0,00	0,00	0,00	0,00	0,00
PSMA2	6,96	6,36	7,12	6,22	6,69	5,82	5,84	5,54	6,09

UBE2V1	7,96	9,55	11,19	7,11	8,60	12,61	8,76	10,16	14,21
LAMC1	15,91	14,85	17,29	10,67	10,51	12,61	9,73	7,39	8,12
F11R	5,97	5,30	8,13	2,67	0,96	2,91	0,97	3,69	2,03
EPCAM	6,96	8,49	6,10	0,89	0,96	1,94	0,97	1,85	2,03
LRRFIP1	4,97	7,42	7,12	7,11	5,73	10,67	8,76	9,24	10,15
WDR1	5,97	5,30	3,05	8,00	9,56	12,61	14,59	10,16	11,16
EWSR1	6,96	8,49	7,12	4,44	4,78	1,94	4,86	3,69	2,03
S100A13	5,97	5,30	6,10	4,44	5,73	4,85	5,84	3,69	5,07
L1CAM	0,99	2,12	1,02	0,00	0,00	0,00	0,00	0,00	0,00
CNBP	5,97	6,36	6,10	7,11	6,69	3,88	4,86	5,54	5,07
RNASET2	4,97	7,42	5,08	0,00	0,00	0,00	0,00	0,00	0,00
PPP2R4	6,96	8,49	7,12	5,33	4,78	5,82	4,86	6,46	8,12
HSP90B1	6,96	5,30	0,00	21,34	23,90	21,35	25,30	19,40	19,28
PSMA7	8,95	6,36	8,13	5,33	7,65	4,85	9,73	7,39	6,09
NUMA1	3,98	4,24	4,07	0,00	0,00	0,00	0,00	0,00	0,00
NCL	18,89	14,85	14,24	24,89	20,07	20,38	24,32	18,47	18,26
RBBP4	6,96	6,36	8,13	5,33	8,60	5,82	5,84	5,54	4,06
CD59	3,98	3,18	5,08	6,22	6,69	6,79	6,81	6,46	7,10
ARHGDIA	6,96	9,55	7,12	8,00	6,69	6,79	3,89	6,46	6,09
DBI	8,95	5,30	8,13	8,00	5,73	6,79	7,78	6,46	7,10
HEXB	1,99	3,18	4,07	8,89	7,65	6,79	6,81	9,24	7,10
PSME2	9,94	9,55	12,20	9,78	9,56	4,85	7,78	5,54	7,10
CBX1	4,97	7,42	7,12	5,33	5,73	5,82	4,86	4,62	6,09
FTH1	3,98	5,30	4,07	0,00	0,00	0,00	0,00	0,92	0,00
CAPRIN1	4,97	6,36	4,07	6,22	4,78	6,79	5,84	5,54	7,10
NOTCH2	4,97	7,42	10,17	1,78	0,96	1,94	1,95	1,85	3,04
TFG	5,97	7,42	5,08	11,56	11,47	11,64	15,57	13,85	13,19
HPRT1	7,96	6,36	7,12	10,67	9,56	7,76	10,70	8,31	8,12

PCSK1N	7,96	8,49	6,10	1,78	1,91	1,94	2,92	3,69	3,04
SSB	7,96	7,42	5,08	5,33	6,69	4,85	3,89	4,62	4,06
TALDO1	8,95	7,42	6,10	4,44	5,73	5,82	5,84	6,46	6,09
VASN	4,97	3,18	5,08	4,44	2,87	4,85	2,92	2,77	4,06
PDIA4	2,98	3,18	4,07	20,45	20,07	23,29	20,43	21,24	24,35
AHCY	6,96	7,42	7,12	11,56	11,47	8,73	14,59	14,78	12,18
CSTB	5,97	6,36	7,12	7,11	7,65	6,79	5,84	8,31	7,10
CLIC3	1,99	4,24	4,07	4,44	3,82	1,94	3,89	3,69	4,06
KHSRP	7,96	10,61	5,08	5,33	5,73	6,79	3,89	6,46	4,06
CMPK1	7,96	4,24	8,13	11,56	7,65	7,76	7,78	6,46	8,12
ACAT2	5,97	6,36	5,08	3,56	2,87	2,91	2,92	4,62	3,04
MAN2A1	4,97	3,18	4,07	0,00	0,96	0,00	0,00	0,00	0,00
ME1	5,97	7,42	7,12	5,33	4,78	6,79	7,78	7,39	7,10
XRCC6	6,96	5,30	6,10	5,33	3,82	2,91	0,97	0,92	1,01
ATP6AP1	4,97	4,24	5,08	8,00	5,73	7,76	5,84	6,46	8,12
SERPINA3	15,91	12,73	16,27	0,00	0,00	0,00	0,00	0,00	0,00
PDCD6IP	7,96	7,42	6,10	12,45	10,51	12,61	9,73	9,24	11,16
HNRNPA3	5,97	5,30	4,07	1,78	0,96	0,97	2,92	2,77	2,03
PCNA	7,96	8,49	9,15	2,67	3,82	5,82	3,89	6,46	6,09
RANBP1	6,96	6,36	6,10	4,44	6,69	5,82	7,78	5,54	6,09
ACTR2	3,98	4,24	6,10	8,00	10,51	11,64	13,62	12,93	13,19
EIF4A1	6,96	6,36	7,12	10,67	9,56	10,67	13,62	12,01	13,19
PDCD5	5,97	4,24	8,13	3,56	4,78	5,82	5,84	3,69	8,12
ISOC1	4,97	7,42	5,08	0,89	0,96	0,97	0,97	0,92	1,01
EPRS	2,98	6,36	4,07	6,22	6,69	4,85	5,84	5,54	5,07
KYNU	6,96	3,18	6,10	3,56	3,82	3,88	1,95	4,62	5,07
RPS12	4,97	4,24	4,07	7,11	1,91	5,82	5,84	5,54	6,09
PPIB	0,99	2,12	4,07	14,22	22,94	17,47	20,43	22,17	20,29

AP2A1	1,99	3,18	3,05	3,56	2,87	0,97	0,97	1,85	3,04
RBBP7	4,97	3,18	6,10	4,44	6,69	4,85	4,86	4,62	4,06
CCT3	6,96	7,42	4,07	9,78	13,38	8,73	8,76	7,39	9,13
PCMT1	4,97	7,42	6,10	5,33	6,69	6,79	6,81	5,54	8,12
RAD23A	7,96	8,49	5,08	3,56	3,82	4,85	3,89	6,46	3,04
NASP	10,94	6,36	8,13	2,67	0,00	0,97	0,00	0,00	0,00
SEC23B	2,98	6,36	5,08	4,44	5,73	4,85	5,84	6,46	6,09
APOA1BP	6,96	6,36	8,13	4,44	5,73	3,88	4,86	5,54	4,06
DSTN	6,96	7,42	7,12	4,44	3,82	4,85	6,81	4,62	8,12
ILF2	4,97	5,30	6,10	2,67	4,78	3,88	3,89	5,54	5,07
SIAE	0,99	1,06	1,02	0,00	0,00	0,00	0,00	0,00	0,00
ARPC5	6,96	7,42	7,12	4,44	6,69	6,79	8,76	6,46	5,07
B4GALT1	5,97	3,18	6,10	3,56	2,87	3,88	0,97	1,85	2,03
ENSA	5,97	7,42	5,08	2,67	2,87	2,91	3,89	3,69	2,03
LGALS3BP	22,87	26,52	24,40	8,00	10,51	9,70	8,76	10,16	7,10
NCAM2	3,98	2,12	2,03	0,00	0,00	0,00	0,00	0,00	0,00
DYNC1I2	5,97	4,24	5,08	5,33	3,82	2,91	4,86	4,62	4,06
GM2A	5,97	4,24	4,07	2,67	1,91	0,97	1,95	1,85	1,01
CCT5	8,95	7,42	5,08	9,78	12,43	7,76	9,73	10,16	5,07
RBM8A	5,97	6,36	6,10	5,33	4,78	2,91	5,84	3,69	5,07
PPP3CA	5,97	4,24	5,08	2,67	1,91	2,91	0,97	2,77	2,03
INHBB	0,99	1,06	1,02	0,00	0,00	0,00	0,00	0,00	0,00
PVRL4	6,96	5,30	5,08	1,78	3,82	1,94	1,95	1,85	1,01
SEC23A	3,98	5,30	5,08	8,89	7,65	5,82	7,78	6,46	8,12
HEBP2	4,97	5,30	6,10	3,56	4,78	4,85	3,89	4,62	4,06
AP1B1	4,97	6,36	5,08	8,89	8,60	7,76	8,76	9,24	8,12
AREG	5,96	4,36	4,10	8,00	6,69	6,79	6,81	6,46	4,06
HINT1	6,96	6,36	5,08	4,44	5,73	5,82	6,81	6,46	7,10

PAFAH1B3	4,97	5,30	6,10	3,56	2,87	1,94	1,95	2,77	2,03
COPG1	3,98	6,36	6,10	8,89	9,56	5,82	8,76	6,46	4,06
RPLP1	4,97	6,36	7,12	5,33	8,60	7,76	8,76	8,31	10,15
EIF4B	4,97	5,30	6,10	6,22	5,73	5,82	5,84	6,46	7,10
NACA	5,97	4,24	5,08	6,22	4,78	4,85	5,84	4,62	3,04
COPB2	3,98	5,30	5,08	3,56	4,78	5,82	3,89	4,62	5,07
USP5	4,97	5,30	7,12	5,33	5,73	5,82	7,78	6,46	8,12
RPSA	1,99	4,24	4,07	8,00	6,69	5,82	4,86	5,54	6,09
LTA4H	4,97	2,12	4,07	4,44	4,78	5,82	3,89	6,46	6,09
PAFAH1B2	4,97	4,24	5,08	3,56	3,82	3,88	3,89	5,54	3,04
GSS	3,98	4,24	4,07	1,78	0,96	2,91	4,86	3,69	4,06
PSMC3	2,98	3,18	7,12	10,67	8,60	7,76	7,78	9,24	8,12
CAPZA2	4,97	4,24	5,08	3,56	4,78	4,85	5,84	4,62	5,07
SUMO2	3,98	5,30	6,10	5,33	3,82	3,88	3,89	3,69	4,06
DUT	1,99	4,24	2,03	3,56	1,91	0,97	1,95	0,92	2,03
TAF15	4,97	5,30	4,07	3,56	1,91	5,82	1,95	2,77	4,06
APEX1	6,96	9,55	7,12	6,22	3,82	4,85	4,86	4,62	5,07
TIMP2	2,98	2,12	2,03	3,56	5,73	1,94	5,84	4,62	5,07
USP14	7,96	3,18	4,07	7,11	5,73	6,79	5,84	7,39	8,12
CNDP2	3,98	3,18	4,07	1,78	1,91	3,88	3,89	4,62	3,04
SLC12A2	4,97	5,30	5,08	0,00	0,00	0,00	0,00	0,00	0,00
ZYX	7,96	4,24	6,10	17,78	20,07	16,50	18,49	20,32	22,32
AP2B1	3,98	4,24	2,03	2,67	4,78	1,94	5,84	1,85	3,04
PLS1	3,98	3,18	5,08	1,78	1,91	2,91	2,92	0,92	1,01
ZNF185	1,99	1,06	2,03	16,00	12,43	18,44	14,59	13,85	16,23
ATP6AP2	2,98	2,12	4,07	5,33	6,69	6,79	4,86	4,62	6,09
PSMB6	3,98	3,18	3,05	3,56	4,78	4,85	5,84	4,62	4,06
DCTPP1	5,97	4,24	4,07	3,56	4,78	6,79	3,89	5,54	6,09

SERPINB6	4,97	3,18	4,07	1,78	0,96	1,94	2,92	2,77	2,03
CAST	4,97	9,55	9,15	5,33	10,51	10,67	14,59	12,01	12,18
RRBP1	2,98	2,12	3,05	15,11	13,38	15,52	11,68	12,93	8,12
NAMPT	3,98	5,30	4,07	8,89	5,73	7,76	10,70	10,16	11,16
38595	3,98	4,24	5,08	5,33	4,78	3,88	3,89	8,31	7,10
ERH	4,97	6,36	4,07	4,44	3,82	2,91	1,95	3,69	5,07
DDX39B	1,99	2,12	5,08	2,67	0,96	2,91	1,95	3,69	4,06
TMPO	4,97	6,36	6,10	2,67	2,87	5,82	1,95	2,77	2,03
EIF4H	7,96	6,36	6,10	8,00	7,65	8,73	6,81	6,46	7,10
ACP1	2,98	2,12	2,03	4,44	4,78	3,88	3,89	2,77	6,09
CRIM1	0,99	1,06	1,02	0,00	0,00	0,00	0,00	0,00	0,00
SNRPD2	3,98	5,30	5,08	3,56	3,82	2,91	3,89	3,69	4,06
STMN1	3,98	6,36	3,05	0,00	0,00	0,00	0,00	0,00	0,00
PGM2	3,98	4,24	6,10	2,67	0,96	1,94	0,97	3,69	2,03
NUTF2	3,98	5,30	7,12	4,44	3,82	3,88	4,86	4,62	7,10
CRIP2	4,97	4,24	5,08	1,78	1,91	0,97	2,92	2,77	1,01
TCEB1	1,99	3,18	2,03	4,44	4,78	3,88	2,92	5,54	5,07
NPC2	3,98	4,24	4,07	3,56	3,82	4,85	3,89	3,69	4,06
IDH1	2,98	3,18	4,07	0,89	0,96	0,97	1,95	1,85	1,01
GNS	0,99	2,12	3,05	8,00	7,65	5,82	6,81	7,39	8,12
LGALS1	10,94	11,67	9,15	18,67	16,25	19,41	23,35	20,32	18,26
COPB1	2,98	3,18	1,02	3,56	4,78	3,88	2,92	2,77	3,04
TCEAL3	2,98	4,24	5,08	6,22	5,73	5,82	3,89	3,69	5,07
PPT1	2,98	2,12	3,05	1,78	2,87	0,00	2,92	1,85	1,01
STARD10	2,98	2,12	3,05	0,00	0,00	0,00	0,00	0,00	0,00
PSMB2	2,98	3,18	2,03	4,44	4,78	4,85	4,86	3,69	6,09
NONO	3,98	2,12	4,07	0,89	2,87	1,94	2,92	0,92	1,01
UBE2I	5,97	4,24	4,07	3,56	0,00	0,97	2,92	1,85	2,03

PTPRG	0,99	3,18	2,03	0,00	0,00	0,00	0,00	0,00	0,00
GNB2L1	2,98	5,30	5,08	10,67	9,56	8,73	9,73	7,39	7,10
PVRL2	4,97	4,24	2,03	0,89	0,96	0,00	0,00	0,00	0,00
CORO1C	3,98	3,18	5,08	11,56	10,51	10,67	9,73	7,39	12,18
PAICS	3,98	2,12	5,08	3,56	1,91	1,94	3,89	2,77	3,04
LAMC2	0,00	0,00	0,00	45,34	47,79	49,48	46,70	46,18	46,67
CALU	1,99	2,12	2,03	17,78	17,21	17,47	15,57	16,62	12,18
HYOU1	2,98	0,00	3,05	22,22	17,21	24,26	21,40	20,32	17,25
PPL	0,99	0,00	1,02	12,45	11,47	13,58	5,84	8,31	8,12
AGR2	3,98	3,18	3,05	17,78	12,43	11,64	10,70	9,24	11,16
ERO1L	0,00	0,00	0,00	8,89	9,56	9,70	10,70	11,08	10,15
PRSS22	0,99	1,06	1,02	16,89	14,34	14,55	13,62	12,93	14,21
TUBA4A	0,00	0,00	0,00	45,34	43,01	40,75	38,92	38,79	33,48
SERPINH1	0,00	1,06	0,00	20,45	19,12	19,41	18,49	16,62	18,26
ERP29	1,99	1,06	2,03	8,00	8,60	6,79	6,81	8,31	8,12
PLOD2	0,00	0,00	0,00	19,56	18,16	23,29	23,35	18,47	22,32
TXNDC5	0,00	1,06	0,00	8,89	10,51	9,70	10,70	7,39	8,12
GOLM1	10,94	7,42	7,12	1,78	1,91	2,91	2,92	2,77	3,04
DBN1	1,99	1,06	1,02	30,23	29,63	30,08	27,24	24,01	23,34
PRDX4	0,00	0,00	0,00	8,89	8,60	8,73	8,76	8,31	9,13
GANAB	0,99	1,06	1,02	8,89	6,69	7,76	6,81	6,46	4,06
VGF	5,97	5,30	5,08	14,22	9,56	7,76	6,81	8,31	7,10
CNPY2	0,99	1,06	1,02	7,11	6,69	6,79	7,78	7,39	6,09
KTN1	0,00	1,06	0,00	8,89	6,69	4,85	6,81	5,54	7,10
GPC4	1,99	2,12	2,03	0,89	0,00	0,00	0,97	1,85	1,01
FSTL3	2,98	2,12	3,05	9,78	10,51	6,79	8,76	6,46	7,10
ANXA2	3,98	2,12	4,07	10,67	9,56	7,76	9,73	10,16	8,12
CLTB	4,97	2,12	1,02	5,33	7,65	4,85	5,84	6,46	3,04

KRT81	0,00	0,00	0,00	3,56	3,82	5,82	5,84	4,62	3,04
OLFML3	0,00	0,00	0,00	13,34	14,34	12,61	13,62	11,08	12,18
FN1	2,98	2,12	5,08	32,00	27,72	23,29	31,13	29,55	24,35
PRSS8	2,98	4,24	3,05	3,56	1,91	0,97	0,97	2,77	2,03
HK1	1,99	1,06	2,03	4,44	3,82	3,88	1,95	0,92	3,04
HSPE1	3,98	1,06	2,03	4,44	5,73	9,70	4,86	7,39	5,07
IDS	0,00	0,00	0,00	2,67	2,87	4,85	1,95	1,85	3,04
FKBP2	0,00	0,00	0,00	1,78	5,73	2,91	1,95	2,77	3,04
ECM1	0,00	0,00	0,00	0,00	0,00	0,00	0,00	0,00	0,00
ERP44	0,00	0,00	0,00	3,56	2,87	1,94	4,86	4,62	3,04
TXNRD1	5,97	3,18	6,10	9,78	10,51	9,70	7,78	10,16	8,12
KIF5B	1,99	3,18	5,08	10,67	7,65	8,73	3,89	7,39	6,09
RCN2	0,00	0,00	0,00	3,56	2,87	2,91	3,89	3,69	2,03
TFPI	0,00	0,00	0,00	2,67	0,96	0,97	0,97	1,85	1,01
ADAM9	2,98	1,06	0,00	4,44	3,82	2,91	3,89	3,69	2,03
LAMB1	0,99	0,00	0,00	0,00	0,00	0,00	0,00	0,00	0,00
PVR	1,99	3,18	3,05	3,56	4,78	6,79	4,86	5,54	7,10
TXNL1	2,98	5,30	3,05	7,11	4,78	4,85	7,78	7,39	9,13
S100A10	0,99	0,00	1,02	5,33	5,73	4,85	4,86	4,62	4,06
COPE	0,99	2,12	3,05	3,56	2,87	3,88	5,84	3,69	4,06
LRPAP1	0,00	0,00	0,00	0,89	0,96	0,00	0,00	0,00	0,00
HEXA	2,98	3,18	1,02	1,78	0,00	1,94	3,89	1,85	2,03
WARS	4,97	6,36	5,08	9,78	12,43	9,70	10,70	13,85	12,18
RTN4	2,98	4,24	0,00	6,22	5,73	5,82	5,84	6,46	8,12
PSMD2	3,98	2,12	3,05	10,67	10,51	7,76	7,78	6,46	6,09
LAD1	0,00	0,00	0,00	3,56	3,82	6,79	3,89	5,54	5,07
IDI1	0,99	1,06	0,00	1,78	3,82	1,94	3,89	2,77	4,06
SEC22B	0,99	1,06	2,03	2,67	2,87	4,85	4,86	3,69	5,07

HMGA1	0,99	1,06	1,02	5,33	3,82	5,82	3,89	4,62	6,09
MYH9	0,99	0,00	0,00	14,22	14,34	8,73	3,89	5,54	3,04
CYCS	0,99	1,06	0,00	4,44	3,82	4,85	2,92	3,69	2,03
ARCN1	3,98	3,18	3,05	3,56	6,69	4,85	4,86	5,54	5,07
SPAG9	0,00	1,06	1,02	6,22	4,78	5,82	6,81	8,31	5,07
GRB2	2,98	4,24	3,05	5,33	2,87	2,91	2,92	3,69	3,04
S100A16	4,97	4,24	4,07	9,78	4,78	3,88	3,89	4,62	3,04
SYTL2	0,00	1,06	1,02	1,78	1,91	0,97	0,97	0,00	1,01
FKBP3	2,98	3,18	4,07	3,56	3,82	2,91	3,89	2,77	3,04
CRKL	1,99	2,12	3,05	2,67	2,87	2,91	3,89	5,54	3,04
TGOLN2	1,99	4,24	3,05	1,78	1,91	1,94	2,92	2,77	3,04
TMOD3	1,99	3,18	4,07	8,00	6,69	7,76	4,86	7,39	8,12
SKP1	2,98	5,30	2,03	6,22	3,82	1,94	7,78	3,69	4,06
SH3BGRL3	3,98	2,12	3,05	6,22	7,65	4,85	4,86	4,62	6,09
OGFR	1,99	3,18	3,05	4,44	3,82	3,88	4,86	3,69	4,06
BID	5,97	4,24	6,10	5,33	3,82	5,82	4,86	4,62	5,07
PSMA6	5,97	4,24	5,08	6,22	4,78	1,94	5,84	6,46	6,09
GPR126	0,00	0,00	0,00	0,00	0,00	0,00	0,00	0,00	0,00
SEC24C	0,00	0,00	1,02	2,67	3,82	2,91	3,89	4,62	4,06
TXNDC12	0,00	0,00	0,00	1,78	0,96	0,00	0,00	0,00	0,00
PTPRJ	0,99	0,00	1,02	0,00	0,00	0,97	0,00	0,00	1,01
KARS	7,96	7,42	9,15	8,00	8,60	8,73	8,76	10,16	9,13
CTSA	0,99	1,06	2,03	1,78	1,91	3,88	2,92	3,69	4,06
DDT	4,97	3,18	4,07	4,44	4,78	4,85	4,86	3,69	5,07
CYR61	2,98	2,12	2,03	5,33	5,73	3,88	3,89	2,77	3,04
PPIC	0,99	1,06	2,03	0,00	0,00	0,97	0,00	0,00	0,00
MET	0,00	0,00	0,00	1,78	1,91	1,94	2,92	2,77	2,03
PPP2CA	1,99	2,12	5,08	5,33	3,82	4,85	6,81	3,69	7,10

S100P	1,99	2,12	2,03	1,78	1,91	1,94	2,92	2,77	2,03
MAPRE1	1,99	3,18	2,03	4,44	5,73	4,85	5,84	7,39	5,07
LPP	0,99	0,00	1,02	0,89	1,91	1,94	0,00	2,77	4,06
DNAJB11	0,00	0,00	0,00	2,67	2,87	2,91	3,89	2,77	3,04
PPM1G	5,97	6,36	5,08	8,89	12,43	11,64	9,73	9,24	9,13
LGMN	0,99	2,12	1,02	1,78	2,87	1,94	2,92	2,77	3,04
CTSL1	0,00	0,00	0,00	0,00	0,96	0,97	1,95	1,85	1,01
IFI30	0,99	2,12	1,02	7,11	5,73	4,85	6,81	5,54	7,10
FUCA1	0,00	0,00	1,02	1,78	0,96	0,97	0,97	0,92	2,03
SEMA3B	0,00	0,00	0,00	1,78	0,00	0,00	0,00	0,92	0,00
SIL1	0,00	0,00	0,00	0,89	1,91	0,97	0,00	0,00	1,01
EPHA2	0,99	0,00	1,02	2,67	2,87	2,91	3,89	3,69	3,04
MCFD2	0,00	0,00	0,00	0,89	0,00	2,91	0,97	4,62	3,04
COX6B1	1,99	1,06	0,00	2,67	2,87	1,94	1,95	2,77	2,03
HARS	3,98	3,18	4,07	3,56	6,69	7,76	8,76	7,39	7,10
KIAA1199	0,00	0,00	0,00	0,00	0,00	0,00	0,00	0,00	0,00
ULBP2	0,00	1,06	1,02	1,78	1,91	4,85	2,92	1,85	5,07
RPLP0	5,97	5,30	5,08	8,00	8,60	11,64	8,76	12,01	13,19
FUCA2	0,99	1,06	1,02	2,67	1,91	0,97	1,95	0,92	1,01
PODXL	0,00	0,00	0,00	0,00	0,00	0,00	0,00	0,00	0,00
HNRNPH3	3,98	4,24	3,05	2,67	2,87	2,91	2,92	3,69	3,04
CHMP4B	2,98	5,30	4,07	2,67	2,87	3,88	2,92	2,77	5,07
HLA-A	2,98	2,12	3,05	4,44	3,82	2,91	2,92	4,62	5,07
ANP32A	7,96	6,36	5,08	7,11	9,56	7,76	8,76	6,46	7,10
SUB1	2,98	6,36	5,08	4,44	4,78	6,79	5,84	8,31	6,09
LDLR	0,99	1,06	1,02	0,89	0,96	1,94	2,92	0,92	2,03
MUC5B	22,87	19,09	19,32	0,00	0,00	0,00	0,00	0,00	0,00
BCAS1	7,96	9,55	11,19	0,00	0,00	0,00	0,00	0,00	0,00

NUCKS1	8,95	7,42	10,17	8,00	8,60	8,73	5,84	7,39	8,12
GNAS	12,93	6,36	7,12	1,78	2,87	0,97	0,97	0,92	2,03
S100A14	8,95	8,49	7,12	8,00	5,73	6,79	6,81	7,39	8,12
CRABP2	6,96	6,36	5,08	3,56	3,82	2,91	3,89	2,77	2,03
GGCT	7,96	4,24	6,10	0,89	0,96	1,94	2,92	2,77	3,04
ANP32B	5,97	6,36	5,08	4,44	6,69	7,76	7,78	7,39	7,10
PDXK	5,97	6,36	5,08	5,33	6,69	3,88	5,84	4,62	5,07
CAND1	4,97	6,36	5,08	6,22	7,65	8,73	6,81	4,62	7,10
LAMB2	5,97	6,36	4,07	0,00	0,00	0,00	0,00	0,00	0,00
GIPC1	5,97	5,30	5,08	3,56	4,78	3,88	1,95	3,69	4,06
GNB1	6,96	4,24	4,07	0,00	0,00	0,97	0,97	0,92	2,03
CD9	4,97	4,24	5,08	2,67	2,87	0,97	1,95	1,85	0,00
CCT6A	3,98	6,36	3,05	5,33	8,60	6,79	5,84	7,39	7,10
ST13	4,97	4,24	4,07	3,56	1,91	3,88	4,86	3,69	4,06
DENR	4,97	4,24	4,07	3,56	3,82	4,85	3,89	2,77	4,06
BLVRA	4,97	4,24	4,07	0,89	0,96	0,97	0,97	0,92	1,01
EPHA4	4,97	4,24	4,07	0,00	0,00	0,00	0,00	0,00	0,00
VPS35	4,97	4,24	4,07	2,67	3,82	2,91	1,95	3,69	6,09
VBP1	5,97	4,24	3,05	6,22	2,87	2,91	1,95	2,77	3,04
GARS	4,97	3,18	5,08	7,11	5,73	4,85	7,78	5,54	7,10
FAM129B	3,98	2,12	7,12	3,56	5,73	5,82	3,89	4,62	4,06
EDF1	2,98	4,24	5,08	2,67	1,91	1,94	1,95	1,85	3,04
PPP1R14B	3,98	4,24	4,07	7,11	3,82	1,94	1,95	1,85	3,04
JUP	3,98	4,24	4,07	3,56	1,91	0,97	1,95	2,77	2,03
TBCA	4,97	4,24	3,05	5,33	4,78	2,91	3,89	5,54	4,06
NAP1L4	3,98	3,18	5,08	7,11	4,78	6,79	6,81	6,46	6,09
GMFB	2,98	2,12	7,12	3,56	4,78	3,88	3,89	5,54	5,07
MMP1	0,00	0,00	0,00	16,00	18,16	18,44	15,57	13,85	16,23

KRT17	0,00	0,00	0,00	13,34	15,29	13,58	13,62	12,93	14,21
PDLIM7	0,00	0,00	0,00	10,67	12,43	12,61	11,68	10,16	12,18
PLIN3	1,99	3,18	2,03	9,78	12,43	10,67	11,68	11,08	13,19
CCT2	2,98	5,30	2,03	11,56	9,56	8,73	7,78	8,31	10,15
ERBB2	0,00	0,00	0,00	10,67	7,65	9,70	9,73	6,46	8,12
PSMC2	2,98	3,18	3,05	5,33	6,69	9,70	5,84	5,54	6,09
FHL2	0,00	0,00	0,00	6,22	6,69	7,76	6,81	5,54	5,07
OLA1	2,98	3,18	2,03	7,11	6,69	6,79	5,84	4,62	7,10
CCT7	1,99	3,18	4,07	4,44	6,69	8,73	6,81	8,31	10,15
EIF3B	3,98	3,18	4,07	4,44	7,65	6,79	7,78	5,54	7,10
EFHD2	0,99	1,06	3,05	5,33	6,69	6,79	7,78	5,54	5,07
TCP1	2,98	3,18	2,03	5,33	6,69	5,82	5,84	6,46	6,09
CCT4	1,99	2,12	3,05	5,33	6,69	5,82	5,84	5,54	6,09
ARPC1B	1,99	4,24	1,02	3,56	7,65	5,82	4,86	8,31	8,12
EIF2S2	1,99	3,18	4,07	5,33	5,73	5,82	6,81	9,24	5,07
UBQLN1	2,98	2,12	3,05	6,22	4,78	5,82	9,73	10,16	8,12
ISG15	1,99	2,12	2,03	4,44	6,69	4,85	3,89	3,69	4,06
TNPO1	2,98	3,18	4,07	5,33	3,82	6,79	5,84	6,46	4,06
EIF3G	0,99	4,24	3,05	3,56	3,82	7,76	2,92	4,62	8,12
CDV3	2,98	2,12	3,05	4,44	5,73	4,85	5,84	3,69	2,03
NUDC	2,98	3,18	5,08	5,33	4,78	4,85	3,89	9,24	9,13
PSMC1	2,98	2,12	2,03	5,33	4,78	4,85	4,86	3,69	3,04
LAMB3	0,00	0,00	0,00	5,33	4,78	4,85	6,81	3,69	2,03
PTGES3	3,98	2,12	3,05	5,33	5,73	3,88	6,81	2,77	3,04
CPA4	0,00	0,00	0,00	6,22	4,78	3,88	1,95	1,85	6,09
PSMC6	1,99	2,12	1,02	4,44	3,82	5,82	3,89	5,54	5,07
EIF3A	0,99	1,06	2,03	4,44	4,78	4,85	4,86	2,77	4,06
COTL1	0,00	1,06	1,02	4,44	5,73	3,88	6,81	3,69	3,04

NAP1L1	1,99	2,12	3,05	5,33	3,82	4,85	3,89	2,77	3,04
ABRACL	1,99	2,12	2,03	2,67	4,78	5,82	2,92	3,69	4,06
AHSA1	1,99	2,12	3,05	3,56	4,78	4,85	3,89	5,54	6,09
SGTA	0,99	1,06	1,02	3,56	6,69	2,91	3,89	5,54	3,04
EIF6	0,99	0,00	1,02	4,44	3,82	4,85	3,89	4,62	6,09
PSMB5	5,97	2,12	2,03	5,33	3,82	3,88	4,86	3,69	6,09
EIF4G1	0,99	1,06	0,00	5,33	3,82	3,88	3,89	1,85	5,07
GLRX3	2,98	1,06	3,05	6,22	1,91	4,85	3,89	4,62	3,04
NRBP1	1,99	2,12	3,05	2,67	4,78	4,85	5,84	3,69	4,06
TMSB4X	4,97	2,12	2,03	3,56	2,87	5,82	4,86	5,54	3,04
AIMP1	0,99	3,18	3,05	3,56	3,82	4,85	1,95	3,69	4,06
RAN	2,98	2,12	3,05	3,56	4,78	3,88	3,89	3,69	6,09
PSME3	0,99	1,06	1,02	3,56	4,78	3,88	1,95	4,62	3,04
BOLA2	2,98	2,12	2,03	4,44	3,82	3,88	2,92	3,69	3,04
VAPB	0,00	0,00	1,02	4,44	3,82	3,88	4,86	3,69	3,04
XPO1	2,98	3,18	3,05	4,44	4,78	2,91	2,92	1,85	1,01
CACYBP	3,98	1,06	2,03	4,44	4,78	2,91	5,84	4,62	4,06
PXN	0,00	0,00	0,00	5,33	2,87	3,88	4,86	4,62	5,07
DDX6	2,98	4,24	3,05	5,33	3,82	2,91	3,89	3,69	5,07
YARS	0,99	1,06	1,02	5,33	3,82	2,91	1,95	3,69	3,04
STAT1	0,00	0,00	0,00	5,33	3,82	2,91	3,89	2,77	3,04
EIF2S1	1,99	3,18	1,02	6,22	3,82	1,94	4,86	2,77	5,07
HSPD1	0,99	1,06	1,02	1,78	2,87	2,91	9,73	5,54	7,10
CNN2	0,99	1,06	2,03	2,67	3,82	3,88	6,81	5,54	7,10
VAT1	0,99	3,18	1,02	2,67	4,78	2,91	6,81	5,54	5,07
PAK2	1,99	3,18	2,03	4,44	2,87	2,91	5,84	4,62	6,09
RNF114	0,00	0,00	0,00	3,56	3,82	2,91	3,89	5,54	7,10
ITGB1	0,00	0,00	0,00	2,67	2,87	3,88	2,92	4,62	7,10

UFM1	2,98	3,18	3,05	3,56	2,87	4,85	3,89	3,69	6,09
TLN1	1,99	3,18	3,05	1,78	1,91	0,00	2,92	5,54	5,07
SERPINB1	0,99	1,06	0,00	2,67	4,78	3,88	3,89	7,39	2,03
RARS	0,99	0,00	1,02	5,33	2,87	2,91	3,89	2,77	6,09
SRM	2,98	2,12	2,03	3,56	2,87	4,85	3,89	3,69	5,07
ARPC5L	2,98	1,06	3,05	2,67	3,82	4,85	3,89	4,62	4,06
Total Spectral counts	7853,92	7866,30	7879,04	7879,83	7967,68	7935,89	7882,56	7929,66	7876,44
