# Supplementary material for: Role of ADAM17 in the non-cell autonomous effects of oncogene-induced senescence
Source: Breast Cancer Res. 2015 Aug 12;17(1):106. doi: 10.1186/s13058-015-0619-7 (PMC4532141; doi:10.1186/s13058-015-0619-7)
Supplement: Additional file 3: Table S2. — Proteins identified by label-free quantitative proteomics secreted through the canonical pathway. Doxy doxycycline. (PDF 44 kb) [file 13058_2015_619_MOESM3_ESM.pdf]

Supplementary Table SII. Proteins identified by label-free quantitative proteomics secreted through the canonical pathway

	Proteins identified secreted (canonical) (Spectral Counts)								
	+Doxy.			-Doxy.					
	MCF7 Tet-Off p95HER2 #1			MCF7 Tet-Off p95HER2 #1			MCF7 Tet-Off p95HER2 #2		
Gene Name	A	B	C	A	B	C	A	B	C
AGR2	0,21	-0,09	-0,14	2,29	1,78	1,69	1,57	1,36	1,63
ALB	-0,15	0,05	0,09	-0,71	0,01	-0,76	-0,31	-0,38	-0,53
B2M	0,14	-0,24	0,07	-0,02	-0,02	-0,23	-0,11	-0,58	-0,17
BTD	0,26	0,05	-0,39	-3,87	-3,87	-3,87	-3,87	-3,87	-3,87
CALR	0,25	0,11	-0,44	1,45	1,56	1,46	1,21	1,36	1,37
CALU	-0,04	0,04	-0,01	2,94	2,9	2,92	2,76	2,85	2,41
CGREF1	0,04	-0,12	0,07	-4,34	-4,34	-4,34	-4,34	-4,34	-4,34
CLU	0,04	0,08	-0,13	-1,98	-1,44	-1,86	-2,04	-2,12	-2,45
COL12A1	-0,24	0,02	0,18	-5,99	-5,99	-5,99	-5,99	-5,99	-5,99
COL18A1	0,06	-0,16	0,09	-4,08	-4,08	-4,08	-4,08	-4,08	-4,08
CPA4	0	0	0	4,44	4,08	3,8	2,9	2,84	4,41
CPE	0,05	0,09	-0,14	-1,2	-1,1	-1,76	-1,44	-1,26	-1,53
CST3	0,17	-0,06	-0,12	-2,54	-1,89	-2,42	-2,12	-2,19	-2,06
CTSD	0,11	-0,06	-0,05	-0,82	-0,71	-0,69	-0,26	-0,49	-0,27
CYR61	0,29	-0,15	-0,2	1,07	1,17	0,64	0,65	0,2	0,32
DKK1	0,07	0,16	-0,27	-2,98	-3,7	-3,69	-3,68	-3,74	-3,64
ECM1	0	0	0	0	0	0	0	0	0
EFEMP1	0,04	-0,03	-0,01	-5,88	-5,88	-5,88	-5,88	-5,88	-5,88
FN1	-0,17	-0,61	0,54	3,13	2,92	2,67	3,09	3,01	2,74
FSTL3	0,12	-0,32	0,15	1,74	1,84	1,23	1,59	1,16	1,29
FUCA2	-0,03	0,04	-0,01	1,16	0,74	-0,06	0,76	-0,11	-0,01

GDF15	-0,13	-0,04	0,16	-0,11	-0,01	-0,08	0,01	0,02	0,07
GGH	0,18	-0,07	-0,13	-5,39	-5,39	-5,39	-3,3	-5,39	-3,26
GNAS	0,54	-0,45	-0,3	-2,13	-1,52	-2,84	-2,84	-2,9	-1,97
GRN	-0,17	0,27	-0,14	0,83	0,53	0,29	0,77	0,69	0,49
GSN	-0,19	-0,03	0,19	-0,55	-0,45	-0,29	-0,35	-0,36	-0,16
HSPG2	0,14	0,23	-0,47	-2,65	-4,64	-4,64	-2,55	-2,61	-2,51
IFI30	-0,37	0,53	-0,35	2,14	1,85	1,62	2,08	1,8	2,14
IGFBP2	-0,15	-0,03	0,16	-1,56	-1,45	-1,58	-1,58	-1,65	-1,3
IGFBP4	0,08	0,11	-0,22	-1,62	-1,92	-1,69	-1,9	-1,97	-2,39
IGFBP5	-0,14	-0,02	0,15	-7,52	-7,52	-7,52	-7,52	-7,52	-7,52
INHBB	-0,03	0,04	-0,01	-2,14	-2,14	-2,14	-2,14	-2,14	-2,14
LAMA5	-0,22	0,24	-0,06	-2,61	-6,3	-2,19	-6,3	-4,27	-4,16
LAMB1	1,04	-1,07	-1,07	-1,07	-1,07	-1,07	-1,07	-1,07	-1,07
LAMB2	0,12	0,21	-0,4	-4,26	-4,26	-4,26	-4,26	-4,26	-4,26
LAMB3	0	0	0	4,23	4,08	4,1	4,57	3,73	2,96
LAMC1	-0,01	-0,11	0,11	-0,57	-0,59	-0,34	-0,7	-1,09	-0,95
LAMC2	0	0	0	7,25	7,32	7,37	7,29	7,28	7,29
LGALS3BP	-0,1	0,11	-0,01	-1,58	-1,2	-1,32	-1,46	-1,25	-1,75
LTBP1	-0,12	-0,03	0,13	-5,47	-5,47	-5,47	-5,47	-5,47	-5,47
MDK	0,03	0,01	-0,05	-1,45	-1,1	-1,33	-1,62	-1,15	-2,47
MMP1	0	0	0	5,76	5,94	5,96	5,72	5,56	5,78
MUC5B	0,16	-0,1	-0,08	-6,11	-6,11	-6,11	-6,11	-6,11	-6,11
NPC2	-0,04	0,05	-0,01	-0,19	-0,09	0,23	-0,07	-0,14	-0,01
NUCB2	0,25	0,13	-0,48	-0,36	-0,26	-0,54	-0,54	-0,31	0,07
OLFML3	0	0	0	5,51	5,61	5,43	5,54	5,25	5,38
PCSK1N	0,08	0,17	-0,29	-1,91	-1,82	-1,8	-1,28	-0,97	-1,22
PRDX4	0	0	0	4,94	4,89	4,91	4,92	4,84	4,97
PRSS22	-0,03	0,04	-0,01	3,7	3,47	3,49	3,39	3,32	3,45

PXDN	-0,06	0,13	-0,08	-2,63	-3,21	-3,71	-2,26	-2,88	-3,13
RNASET2	-0,22	0,33	-0,19	-4,35	-4,35	-4,35	-4,35	-4,35	-4,35
SEMA3B	0	0	0	2,79	0	0	0	2,03	0
SEMA3C	0,12	-0,1	-0,03	-4,34	-4,26	-6,33	-4,25	-6,33	-6,33
SERPINA3	0,09	-0,23	0,12	-5,67	-5,67	-5,67	-5,67	-5,67	-5,67
SIAE	-0,03	0,04	-0,01	-2,14	-2,14	-2,14	-2,14	-2,14	-2,14
SPINT1	0,15	0,05	-0,23	-0,41	-0,1	-0,69	-0,29	0,04	-0,23
STC2	-0,09	0	0,08	-2,36	-2,42	-3,05	-2,58	-2,66	-2,99
TFF1	-0,16	0,06	0,08	-1,71	-1,61	-1,47	-1,84	-1,54	-1,41
TFF3	0,02	-0,07	0,05	-5,82	-5,82	-5,82	-5,82	-5,82	-5,82
TIMP1	0,02	-0,17	0,13	-0,14	-0,04	-0,19	-0,29	-0,17	-0,33
TIMP2	0,29	-0,15	-0,2	0,53	1,17	-0,26	1,2	0,88	1
VGF	0,12	-0,04	-0,1	1,34	0,78	0,49	0,31	0,58	0,36
