# Supplementary material for: Role of ADAM17 in the non-cell autonomous effects of oncogene-induced senescence
Source: Breast Cancer Res. 2015 Aug 12;17(1):106. doi: 10.1186/s13058-015-0619-7 (PMC4532141; doi:10.1186/s13058-015-0619-7)
Supplement: Additional file 4: Table S3. — Proteins identified by label-free quantitative proteomics with transmembrane or GPI-anchored domains and mRNA levels by transcriptomic analysis. Doxy doxycycline, GPI glycophosphatidylinositol. (PDF 52 kb) [file 13058_2015_619_MOESM4_ESM.pdf]

Supplementary Table SIII. Proteins identified by label-free quantitative proteomics with transmembrane or GPI-anchored domains and mRNA levels by transcriptomic analysis.

	Proteins with transmembrane or GPI domains (Spectral Counts)									mRNA (Log2FC)		
	+Doxy.			-Doxy.						+Doxy.	-Doxy.	
	MCF7 Tet-Off p95HER2 #1			MCF7 Tet-Off p95HER2 #1			MCF7 Tet-Off p95HER2 #2			MCF7 Tet-Off p95HER2 #1	MCF7 Tet-Off p95HER2 #1	MCF7 Tet-Off p95HER2 #2
Gene Name	A	B	C	A	B	C	A	B	C			
ADAM9	2,98	1,06	0,00	4,44	3,82	2,91	3,89	3,69	2,03	-0,01	1,12	1,21
ALCAM	26,85	27,58	23,39	6,22	7,65	9,70	3,89	6,46	7,10	-0,02	0,37	0,43
APLP2	10,94	7,42	7,12	6,22	6,69	4,85	5,84	2,77	6,09	-0,05	0,82	0,99
APP	26,85	26,52	31,52	14,22	11,47	11,64	13,62	12,01	12,18	-0,42	0,07	0,46
AREG	5,96	4,36	4,10	8,00	6,69	6,79	6,81	6,46	4,06	-0,11	0,82	1,51
ATP6AP1	4,97	4,24	5,08	8,00	5,73	7,76	5,84	6,46	8,12	0,13	0,33	0,26
ATP6AP2	2,98	2,12	4,07	5,33	6,69	6,79	4,86	4,62	6,09	-0,24	-0,12	0,02
B4GALT1	5,97	3,18	6,10	3,56	2,87	3,88	0,97	1,85	2,03	0,11	0,52	0,39
BCAM	14,92	15,91	14,24	0,00	0,00	0,00	0,00	0,00	0,00	0,42	0,13	-0,11
CADM1	3,98	4,24	4,07	0,00	0,00	0,00	0,00	0,00	0,00	-0,02	-0,60	-0,54
CD59	3,98	3,18	5,08	6,22	6,69	6,79	6,81	6,46	7,10	0,11	2,14	2,11
CD9	4,97	4,24	5,08	2,67	2,87	0,97	1,95	1,85	0,00	-0,19	0,07	0,09
CDH1	20,88	20,15	21,35	8,89	10,51	11,64	8,76	10,16	9,13	-0,31	0,39	0,35
CELSR2	22,87	25,46	23,39	0,00	0,00	0,00	0,00	0,00	0,00	0,09	-0,43	-0,56
CLIC1	13,92	14,85	17,29	19,56	21,03	21,35	25,30	22,17	22,32	-0,02	0,72	0,65
CLIC3	1,99	4,24	4,07	4,44	3,82	1,94	3,89	3,69	4,06	-0,02	-1,66	-1,66
CLSTN1	43,75	39,25	37,62	14,22	14,34	16,50	14,59	16,62	16,23	0,16	0,67	0,47
CRIM1	0,99	1,06	1,02	0,00	0,00	0,00	0,00	0,00	0,00	-0,29	0,59	0,99
DAG1	9,94	11,67	11,19	2,67	1,91	2,91	1,95	1,85	3,04	-0,05	0,07	-0,04

DDR1	5,97	5,30	5,08	0,00	0,00	0,00	0,00	0,92	0,00	0,04	0,62	0,48
EPCAM	6,96	8,49	6,10	0,89	0,96	1,94	0,97	1,85	2,03	-0,13	-0,17	-0,09
EPHA2	0,99	0,00	1,02	2,67	2,87	2,91	3,89	3,69	3,04	0,14	2,92	2,75
EPHA4	4,97	4,24	4,07	0,00	0,00	0,00	0,00	0,00	0,00	-0,08	0,08	0,07
EPHB4	8,95	11,67	8,13	0,00	0,00	0,00	0,00	0,00	0,00	0,14	-0,19	-0,11
F11R	5,97	5,30	8,13	2,67	0,96	2,91	0,97	3,69	2,03	0,06	0,52	0,41
FREM2	0,99	4,24	3,05	0,00	0,00	0,00	0,00	0,00	0,00	-0,16	-3,43	-3,65
GFRA1	5,97	5,30	6,10	0,00	0,00	0,00	0,00	0,00	0,00	0,31	-2,75	-2,88
GLG1	5,97	7,42	7,12	1,78	1,91	0,97	1,95	2,77	2,03	-0,06	0,19	0,26
GOLM1	10,94	7,42	7,12	1,78	1,91	2,91	2,92	2,77	3,04	0,11	0,49	0,36
GPC1	5,97	9,55	8,13	0,00	0,00	0,00	0,00	0,00	0,00	0,13	0,21	0,10
GPC4	1,99	2,12	2,03	0,89	0,00	0,00	0,97	1,85	1,01	-0,06	1,42	1,43
GPR126	0,00	0,00	0,00	0,00	0,00	0,00	0,00	0,00	0,00	-0,25	0,58	0,58
HLA-A	2,98	2,12	3,05	4,44	3,82	2,91	2,92	4,62	5,07	0,15	1,44	1,33
ITGB1	0,00	0,00	0,00	2,67	2,87	3,88	2,92	4,62	7,10	-0,04	0,96	0,95
KTN1	0,00	1,06	0,00	8,89	6,69	4,85	6,81	5,54	7,10	-0,07	0,44	0,48
L1CAM	0,99	2,12	1,02	0,00	0,00	0,00	0,00	0,00	0,00	0,21	-0,50	-0,66
LDLR	0,99	1,06	1,02	0,89	0,96	1,94	2,92	0,92	2,03	-0,01	0,79	0,64
LSR	15,91	16,97	17,29	11,56	13,38	12,61	14,59	12,01	14,21	0,17	0,80	0,40
LYPD3	14,92	14,85	15,25	6,22	6,69	5,82	5,84	5,54	7,10	0,19	0,93	0,71
MAN2A1	4,97	3,18	4,07	0,00	0,96	0,00	0,00	0,00	0,00	-0,10	0,51	0,62
MET	0,00	0,00	0,00	1,78	1,91	1,94	2,92	2,77	2,03	-0,10	2,19	2,16
NCAM2	3,98	2,12	2,03	0,00	0,00	0,00	0,00	0,00	0,00	-0,14	-1,31	-1,24
NEO1	10,94	15,91	12,20	0,00	0,00	0,00	0,00	0,00	0,00	-0,03	-0,15	-0,18
NOTCH2	4,97	7,42	10,17	1,78	0,96	1,94	1,95	1,85	3,04	-0,14	-0,03	0,07
NRCAM	18,89	16,97	14,24	0,00	0,00	0,00	0,00	0,00	0,00	-0,08	-0,54	-0,44
NRP1	8,95	7,42	9,15	3,56	0,96	2,91	0,97	1,85	2,03	0,02	0,26	0,42
PODXL	0,00	0,00	0,00	0,00	0,00	0,00	0,00	0,00	0,00	0,02	1,21	1,39

PRSS8	2,98	4,24	3,05	3,56	1,91	0,97	0,97	2,77	2,03	0,21	1,16	0,86
PTPRF	42,76	56,22	49,82	0,00	0,00	0,00	0,00	0,00	0,00	-0,01	-0,04	-0,01
PTPRG	0,99	3,18	2,03	0,00	0,00	0,00	0,00	0,00	0,00	-0,05	0,05	-0,11
PTPRJ	0,99	0,00	1,02	0,00	0,00	0,97	0,00	0,00	1,01	-0,03	0,26	0,48
PTPRK	15,91	15,91	18,30	1,78	0,96	0,97	0,97	1,85	1,01	0,06	-0,13	-0,04
PVR	1,99	3,18	3,05	3,56	4,78	6,79	4,86	5,54	7,10	0,19	1,25	1,06
PVRL2	4,97	4,24	2,03	0,89	0,96	0,00	0,00	0,00	0,00	0,29	0,34	0,01
PVRL4	6,96	5,30	5,08	1,78	3,82	1,94	1,95	1,85	1,01	-0,27	0,80	0,64
QSOX1	33,81	32,88	29,49	60,45	57,35	57,25	54,48	54,49	55,80	-0,14	2,33	2,23
RRBP1	2,98	2,12	3,05	15,11	13,38	15,52	11,68	12,93	8,12	0,11	0,46	0,54
RTN4	2,98	4,24	0,00	6,22	5,73	5,82	5,84	6,46	8,12	0,08	0,37	0,49
SDC4	11,93	13,79	13,22	8,89	10,51	9,70	11,68	10,16	11,16	0,10	1,54	1,56
SEC22B	0,99	1,06	2,03	2,67	2,87	4,85	4,86	3,69	5,07	-0,05	-0,02	0,19
SLC12A2	4,97	5,30	5,08	0,00	0,00	0,00	0,00	0,00	0,00	-0,15	-0,82	-0,76
SLC3A2	9,94	11,67	9,15	11,56	12,43	11,64	13,62	12,01	10,15	-0,03	-0,14	-0,30
ST14	9,94	8,49	13,22	4,44	3,82	6,79	3,89	4,62	4,06	0,01	0,65	0,43
TGOLN2	1,99	4,24	3,05	1,78	1,91	1,94	2,92	2,77	3,04	-0,06	0,33	0,26
TMPO	4,97	6,36	6,10	2,67	2,87	5,82	1,95	2,77	2,03	0,09	-0,95	-1,04
ULBP2	0,00	1,06	1,02	1,78	1,91	4,85	2,92	1,85	5,07	-0,14	2,50	2,28
VAPB	0,00	0,00	1,02	4,44	3,82	3,88	4,86	3,69	3,04	0,10	0,03	-0,14
VASN	4,97	3,18	5,08	4,44	2,87	4,85	2,92	2,77	4,06	0,07	1,26	1,23
Total Spectral counts	550,90	569,72	557,23	303,14	292,48	311,46	292,85	294,61	304,38			
