# Supplementary material for: Role of ADAM17 in the non-cell autonomous effects of oncogene-induced senescence
Source: Breast Cancer Res. 2015 Aug 12;17(1):106. doi: 10.1186/s13058-015-0619-7 (PMC4532141; doi:10.1186/s13058-015-0619-7)
Supplement: Additional file 6: Table S4. — List of confirmed proteins from label-free proteomic analysis. (PDF 36 kb) [file 13058_2015_619_MOESM6_ESM.pdf]

Supplementary Table SIV. List of confirmed proteins from label-free proteomic analysis.

Transmembrane protein	Molecular weight	Reference
Met	145-175 kDa	[33]
sMet	80 kDa	
APP	110-135 kDa	[34]
sAPP	115 kDa	
EpCAM	31-39 kDa	[35]
sEpCAM	33 kDa	
EphB4	110 kDa	[36]
sEphB4	60 kDa	
DDR1	120 kDa	[14]
sDDR1	60 kDa	
