# Supplementary material for: Role of ADAM17 in the non-cell autonomous effects of oncogene-induced senescence
Source: Breast Cancer Res. 2015 Aug 12;17(1):106. doi: 10.1186/s13058-015-0619-7 (PMC4532141; doi:10.1186/s13058-015-0619-7)
Supplement: Additional file 7: Table S5. — Proteins identified by label-free quantitative proteomics in MCF7 Tet-Off p95HER2 shNT and shADAM17. Doxy doxycycline, GPI glycophosphatidylinositol. (PDF 44 kb) [file 13058_2015_619_MOESM7_ESM.pdf]

Supplementary Table SV. Proteins identified by label-free quantitative proteomics in MCF7 Tet-Off p95HER2 shNT and shADAM17

	Proteins with transmembrane or GPI domains (Spectral Counts) -Doxy.								
	MCF7 Tet-Off p95HE2R shA17 #1			MCF7 Tet-Off p95HER2 shA17 #2			MCF7 Tet-Off p95HER2shNT		
Gene Name	A	B	C	A	B	C	A	B	C
ADAM9	10	10	9	12	17	19	10	10	13
ALCAM	5	6	6	5	3	4	9	12	9
APLP2	13	8	11	2	5	8	16	8	10
APP	8	13	14	10	9	12	16	18	17
AREG	6	5	5	11	7	9	11	15	10
ATP6AP1	15	13	17	25	21	19	16	17	17
ATP6AP2	8	6	7	11	9	5	7	7	6
B4GALT1	10	8	8	3	5	4	8	8	6
BCAM									
CADM1									
CD59	4	5	7	10	13	15	6	8	9
CD9	3	4	5	1	3	1	3	4	4
CDH1	11	8	8	19	17	17	16	13	12
CELSR2									
CLIC1	25	23	24	34	35	36	21	24	24
CLIC3	8	6	7	10	9	4	4	6	6
CLSTN1	24	23	25	27	14	20	24	27	23
CRIM1									
DAG1	8	9	6	15	16	15	11	10	13
DDR1									
EPCAM	2	2	3	1	4	1	5	2	4
EPHA2	1	3	1	0	0	0	3	5	2

EPHA4									
EPHB4									
F11R	3	2	2	1	3	3	4	3	4
FREM2									
GFRA1									
GLG1	4	4	6	3	3	3	2	3	3
GOLM1	9	10	8	20	18	17	10	11	13
GPC1	3	2	2	2	0	0	4	3	2
GPC4									
GPR126	0	1	1	1	3	1	2	2	1
HLA-A	1	2	1	1	1	3	2	4	4
ITGB1	1	5	3	2	3	3	2	3	3
KTN1	6	6	6	6	3	4	5	8	6
L1CAM									
LDLR	3	2	2	3	3	4	4	3	4
LSR	22	18	18	28	31	40	18	20	21
LYPD3	54	34	28	32	34	28	40	28	24
MAN2A1									
MET	1	3	2	0	0	0	4	2	6
NCAM2									
NEO1									
NOTCH2	6	4	4	9	12	13	5	1	5
NRCAM									
NRP1	9	8	10	5	7	5	8	11	12
PODXL									
PRSS8	2	0	0	0	0	0	1	1	1
PTPRF									
PTPRG									

PTPRJ	1	1	1	5	5	5	3	3	2
PTPRK	3	1	0	2	4	3	3	1	2
PVR	10	10	9	15	21	20	12	11	15
PVRL2									
PVRL4									
QSOX1	89	98	99	130	132	98	94	100	97
RRBP1	21	21	17	28	30	35	29	28	35
RTN4	8	8	8	18	10	9	9	11	10
SDC4	9	9	9	18	20	20	19	19	18
SEC22B	3	3	3	2	3	4	2	4	3
SLC12A2									
SLC3A2	13	13	12	11	13	8	11	16	11
ST14	8	8	7	3	4	3	10	12	11
TGOLN2	3	6	3	7	8	8	5	6	5
TMPO	10	9	12	10	12	11	9	8	10
ULBP2									
VAPB	6	6	5	6	8	7	5	3	1
VASN	10	11	12	20	18	20	20	24	21
Total Spectral counts	479	457	453	584	596	564	528	543	535
